# Short-term treatment of golden retriever muscular dystrophy (GRMD) dogs with rAAVrh74.MHCK7.*GALGT2* induces muscle glycosylation and utrophin expression but has no significant effect on muscle strength

**DOI:** 10.1371/journal.pone.0248721

**Published:** 2021-03-26

**Authors:** Paul T. Martin, Deborah A. Zygmunt, Anna Ashbrook, Sonia Hamilton, Davin Packer, Sharla M. Birch, Amanda K. Bettis, Cynthia J. Balog-Alvarez, Lee-Jae Guo, Peter P. Nghiem, Joe N. Kornegay

**Affiliations:** 1 Center for Gene Therapy, Abigail Wexner Research Institute, Nationwide Children’s Hospital, Columbus, Ohio, United States of America; 2 Department of Pediatrics, The Ohio State University College of Medicine, Columbus, Ohio, United States of America; 3 Neuroscience Undergraduate Program, The Ohio State University, Columbus, Ohio, United States of America; 4 Neuroscience Graduate Program, The Ohio State University, Columbus, Ohio, United States of America; 5 Department of Veterinary Integrative Biosciences, College of Veterinary Medicine and Biomedical Sciences, Texas A&M University, College Station, TX, United States of America; University of Minnesota Medical School, UNITED STATES

## Abstract

We have examined the effects of intravenous (IV) delivery of rAAVrh74.MHCK7.*GALGT2* in the golden retriever muscular dystrophy (GRMD) model of Duchenne Muscular Dystrophy (DMD). After baseline testing, GRMD dogs were treated at 3 months of age and reassessed at 6 months. This 3–6 month age range is a period of rapid disease progression, thus offering a relatively short window to establish treatment efficacy. Measures analyzed included muscle AAV transduction, *GALGT2* transgene expression, *GALGT2*-induced glycosylation, muscle pathology, and muscle function. A total of five dogs were treated, 4 at 2x10^14^vg/kg and one at 6x10^14^vgkg. The 2x10^14^vg/kg dose led to transduction of regions of the heart with 1–3 vector genomes (vg) per nucleus, while most skeletal muscles were transduced with 0.25–0.5vg/nucleus. *GALGT2*-induced glycosylation paralleled levels of myofiber vg transduction, with about 90% of cardiomyocytes having increased glycosylation versus 20–35% of all myofibers across the skeletal muscles tested. Conclusions from phenotypic testing were limited by the small number of dogs. Treated dogs had less pronounced fibrosis and overall lesion severity when compared to control groups, but surprisingly no significant changes in limb muscle function measures. *GALGT2*-treated skeletal muscle and heart had elevated levels of utrophin protein expression and *GALGT2*-induced expression of glycosylated α dystroglycan, providing further evidence of a treatment effect. Serum chemistry, hematology, and cardiac function measures were largely unchanged by treatment. Cumulatively, these data show that short-term intravenous treatment of GRMD dogs with rAAVrh74.MHCK7.*GALGT2* at high doses can induce muscle glycosylation and utrophin expression and may be safe over a short 3-month interval, but that such treatments had only modest effects on muscle pathology and did not significantly improve muscle strength.

## Introduction

Genetic treatments for muscular dystrophy have frequently been tested in mouse models of the disease, but the increased size and severity of canine muscular dystrophy models provides several advantages over small animal models. These include a better understanding of the scalability of treatments, safety assessment, and their therapeutic effectiveness [1]. Considerable emphasis has been placed on Duchenne Muscular Dystrophy (DMD), which is the most common genetic form of the muscular dystrophies, occurring at a frequency of about 1 in 5,000 boys due to loss of function mutations in the dystrophin gene (*DMD*) on the X chromosome [2–4]. An inactivating point mutation in exon 23 of the *Dmd* gene in *mdx* mice leads to dystrophin deficiency that models molecular aspects of DMD in skeletal and cardiac muscle [5]. However, while *mdx* mice show severe limb muscle damage in the 3^rd^ to 6^th^ postnatal weeks, this dystrophy then stabilizes in most muscles, with aged mice having little overall muscle wasting other than in the diaphragm [6, 7]. A similar slow progression is seen in the cardiomyopathy phenotype, where evidence of cardiac histopathology and altered cardiac function are not particularly evident at younger ages [8–10]. While a large number of genetic modifiers have been suggested to account for these muted *mdx* disease phenotypes, dystrophin deficiency [11–17], in and of itself, appears to lead to decidedly less severe disease in mice than is found in humans.

We identified a canine DMD model, termed golden retriever muscular dystrophy (GRMD) in the 1980s [18] and, together with others, have defined key phenotypic features in affected dogs that tend to mirror stereotypical aspects of human DMD [18, 19]. Dystrophin deficiency in GRMD dogs arises due to a *DMD* gene mutation that leads to skipping of exon 7 and an out-of-frame transcript with a stop codon in the amino terminal domain [20]. GRMD dogs have a more severe phenotype than *mdx* mice, with signs progressing markedly between 3 and 6 months of age, corresponding to the decline in function at 5–10 years in DMD [19]. In keeping with the variable clinical phenotype seen in DMD and largely unlike *mdx* mice, GRMD dogs have variable disease progression, likely due in part to the fact that dogs are outbred and prone to express different alleles of modifier genes. Because of this phenotypic variability, in designing GRMD preclinical trials, it is best to judge relative functional loss or gain, comparing the mean differences between baseline and termination outcome parameters for individual dogs [19, 21]. The translational value of the GRMD and other canine muscular dystrophy models has been supported by preclinical studies across various treatment modalities, including gene therapies that utilize AAV vectors [19, 22–32].

The *GALGT2* gene encodes a β1,4 GalNAc glycosyltransferase that is localized to the neuromuscular and myotendinous junctions in adult skeletal muscle [33, 34]. *GALGT2* overexpression can protect both wild type and dystrophic mouse skeletal myofibers from injury in mouse models, including the *mdx* model for DMD, the *dy*^*W*^ model for Congenital Muscular Dystrophy type 1A (MDC1A), the *Sgca* model for Limb Girdle Muscular Dystrophy 2D (LGMD2D), and the *FKRP*^*P448L*^ model for Limb Girdle Muscular Dystrophy 2I (LGMD2I) [35–38]. *GALGT2* overexpression stimulates a multifactorial therapeutic mechanism that involves: 1. Glycosylating α dystroglycan (DG) with the Cytotoxic T cell (CT) glycan, 2. Increasing the ectopic overexpression of synaptic extracellular matrix (ECM) proteins, including laminin α2, α4, and α5, and agrin, 3. Increasing the amount of ECM binding to αDG, 4. Inducing overexpression of membrane-stabilizing transmembrane proteins, including DG and integrin α7β1, and 5. Inducing the overexpression of DG-associated membrane-stabilizing filamentous (F)-actin-binding proteins, including dystrophin, utrophin, and plectin1 [35–39]. Expression of a number of surrogate genes and proteins stimulated by *GALGT2* overexpression, like *GALGT2* itself, is normally confined to the neuromuscular junction (NMJ) and the myotendinous junction (MTJ) in adult muscle, but in early postnatal muscle they are spread along the entirety of the sarcolemmal membrane [17, 33, 40–46]. Thus, by drawing such proteins into an extrasynaptic expression pattern in adult muscle, *GALGT2* is essentially re-creating the molecular environment normally seen in a young muscle, a sort of “fountain of youth” therapy. Here, we have tested, for the first time, *GALGT2* (also *B4GALNT2*) gene therapy in GRMD dogs.

## Materials and methods

### Animals

All dogs in this study were produced in a GRMD colony at Texas A&M University. Conception rate and gestation in GRMD are not impaired. Breeding affected males to obligate carriers produces an expected 1:1 ratio of affected to unaffected dogs (25% affected males, 25% affected homozygous females, 25% obligate female carriers, and 25% normal males). Notably, based on previous skeletal muscle function data [47], hemizygous male and homozygous female dogs have a similar disease phenotype, so data were not assessed separately based on gender. The GRMD genotype was suspected based on elevated serum creatine kinase and confirmed by genotyping, as previously described [48]. We studied a total of six GRMD (5 treated, one control) dogs; 2 hemizygous male and 3 homozygous female treated and 1 hemizygous control).

Dogs were cared for and assessed according to principles outlined in the National Research Council’s Guide for the Care and Use of Laboratory Animals. Studies were approved by the Institutional Animal Care and Use Committee (IACUC) at Texas A&M University through IACUC protocol 2016–0048, Glycosyltransferase Therapies for Myopathies. They were observed daily by medical staff (with multiple observations per day) for general and safety issues, including body weight, appetite, hydration, demeanor, overall activity, urination, defecation, general physical condition, and physical examination (when deemed necessary). Dogs underwent general anesthesia for muscle biopsies and functional studies. Analgesics were used to assure that postoperative discomfort and pain were minimized. The six GRMD dogs were euthanized at the end of study (~ 6 months of age) with Beuthanasia (390 mg pentobarbital sodium/ml and 50 mg phenytoin sodium/ml); 1 ml/10 lbs body weight IV. This method is consistent with recommendations of the AVMA Panel on Euthanasia. Normal and carrier dogs produced in the same litters were adopted after neutering/spaying.

Results from *GALGT2*-treated dogs were compared to data from a group of untreated control dogs, including three from another blinded study, plus a historical dataset of 8 GR and 10 untreated GRMD dogs [37]. For myocardial T1 mapping on cardiac magnetic resonance (CMR) imaging, we also compared data to 8 wild type GR dogs from an unrelated natural history study. The only high dose treated dog (Nani) was interpreted separately for both skeletal muscle and cardiac function/CMR studies. For histopathology measures, all tissues were cut and stained for this study from biopsies taken from untreated GR, untreated GRMD, and the 5 treated GRMD dogs, along with 7 untreated GRMD and 3 GR samples from previous studies.

### Recombinant Adeno Associated Virus (AAV) production

All AAV vectors were made and purified by the Viral Vector Core at Nationwide Children’s Hospital (NCH). Because human proteins can sometimes induce unwanted immune responses in GRMD dogs [49], we chose to express the canine *GALGT2* gene for these experiments rather than the human *GALGT2* gene. rAAVrh74.MHCK7.*GALGT2* (canine) was made using the triple transfection method in HEK293 cells as previously described [50]. AAV was purified using iodixanol density separation and anion exchange chromatography as previously described [50, 51]. AAV was titered using a supercoiled DNA standard.

### Intravenous dosing of rAAVrh74.MHCK7.*GALGT2*

Immunosuppression via prednisone (1 mg/kg, PO daily) was initiated on Day -7 (one week prior to dosing with the test article, rAAVrh74.MHCK7.*GALGT2)*, and was maintained until study Day 28 for a total of 5 weeks duration. Additionally, antihistamine (diphenhydramine, 2 mg/kg) was administered subcutaneously 30 minutes before dosing on Day 0. The rAAVrh74.MHCK7.*GALGT2* construct was diluted in lactated ringer’s solution and given through either the cephalic or saphenous vein by continuous infusion at 1.0 +/- 0.5 ml/min, not exceeding 10 ml/kg/hr, with a target infusion time of 20 +/- 5 minutes. Investigators were not blinded to the treated dogs. Four GRMD dogs were dosed with 2x10^14^vg/kg rAAVrh74.MHCK7.*GALGT2(canine)*, and one dog (Nani) was dosed with 6x10^14^vg/kg rAAVrh74.MHCK7.*GALGT2(canine)*. One additional GRMD dog was untreated. Dogs were monitored for adverse reactions throughout the perfusion protocol and for 30 minutes after completion. No adverse reactions during infusion were observed.

Biopsies and functional studies were completed prior to treatment (baseline; day 0) and on days 45 and 90 after test article administration. For all studies, dogs were premedicated with acepromazine maleate (0.02 mg/kg), butorphanol tartrate (0.4 mg/kg), and atropine sulfate (0.04 mg/kg), masked by sevoflurane for anesthetic induction, and then intubated and maintained with sevoflurane. Tibiotarsal joint (TTJ) torque and eccentric contraction decrement (ECD) studies were done on the left pelvic limb at baseline followed under the same anesthetic by open surgical biopsy of the cranial sartorius (CS) and vastus lateralis (VL) muscles at baseline. At Day 45, the left biceps femoris muscle was biopsied. Functional studies were repeated on the right pelvic limb at Day 90 followed immediately by necropsy.

### Tibiotarsal joint (TTJ) torque and eccentric contraction decrement (ECD) studies

Measurements of TTJ torque and ECD were made with a rapid-response servomotor/force transducer (model 310B LR, Aurora Scientific, Inc., Aurora, Ontario, CANADA) controlled by a personal computer using custom LabView software (National Instruments, Austin, TX USA) using established methods [47, 52, 53]. Briefly, supramaximal 150V, 100 μsec pulses were applied percutaneously (Model S48 Solid State Square Wave Stimulator, Grass Instruments, Quincy, MA, USA) in a 1½ sec tetanic run of 75 pulses (50/sec) to the fibular (peroneal; flexion) and tibial (extension) nerves. The site of contact for the paw with the lever (moment arm) was estimated to be 75% of the distance between the point of the hock and the distal digit. Torque (Newton [N]-meters [m]) was divided by the moment arm (m) to convert to force (N).

Eccentric contractions were induced by percutaneously stimulating the fibular nerve using square wave pulses of 100 μsec duration in a tetanic run for 1 sec at a frequency of 50 Hz while simultaneously extending the TTJ with a servomotor (Aurora Scientific) [47, 53, 54]. Thus, the muscles of the cranial tibial compartment were repeatedly stretched to induce mechanical damage. Three sets of 10 stretches for a total of 30, each set separated by 4 min, were performed. Contraction-induced injury was quantified by the force (torque) deficit (Fd) using the following equation: Fd = (Maximal isometric tetanic force [Po] before stretch—Po after stretch) / Po before stretch X 100.

### 6-minute-walk test (6MWT)

Dogs were assessed at 3 (baseline, pretreatment) and 6 months of age using a published protocol [55]. Briefly, a 6MWT course was laid out in the hallway of a building used for veterinary care. Assessment was performed at a consistent time during the day when there was minimal other activity that could be a distraction. Cones were placed 8.8 meters apart, such that the dog would repeatedly circle the cones over a period of 6 minutes. Body weight, height at the withers, length from the occiput to the rump, and heart and respiratory rate were recorded before and/or after each test. Two leashes of equal length were crossed around the neck and one each per thoracic limb to create a body harness. Dogs were leash walked by the same technician, while another technician sat at the far end of the course. A timer was set to six minutes and the number of laps completed was recorded. Dogs were encouraged to walk by verbal prompts and given food treats. A slight leash tug was applied if the dog stopped. The number of times a dog rested was recorded, while time continued to elapse on the timer. If the dog stopped to urinate or defecate, the timer was stopped and resumed when the dog continued to walk.

### Cardiac studies

The cardiac evaluations were performed at pre-treatment, day 45, and day 90 of the study. At each time point, dogs were transported and rested in a quiet room at least 10 minutes before the procedure. Physical exams were performed to assess the health status of the dogs and resting heart rate, respiratory rate, and blood pressure were recorded. Dogs were premedicated and placed under general anesthesia using the same anesthetic protocol detailed above. Echocardiography measures were done using a GE Vivid E9 (GE Healthcare) ultrasound machine using an established protocol [56]. All echocardiographic recordings were later imported into a GE EchoPAC workstation (GE Healthcare) for data analysis. Left ventricular chamber size and systolic function were assessed [57, 58]. Left ventricular end-diastolic volume (LVEDV), end-systolic volume (LVESV), ejection fraction (EF), and stroke volume (SV) were measured and calculated from the right parasternal long-axis view using the modified single-plane Simpson’s method [59]. All measurements were obtained from an average of at least 3 to 5 cardiac cycles. After the echocardiographic scans, the dogs were involved in a separate cardiac stress pilot study (unpublished data) and then recovered from anesthesia.

The CMR scans were performed at least 24 hours apart from echocardiography. Dogs were placed under general anesthesia using the same anesthetic protocol. The CMR scans were performed on a 3-Tesla magnetic resonance imaging machine (Siemens 3T Magnetom Verio, Siemens Medical Solutions) with dogs in a dorsal recumbent position. A cardiac dedicated surface coil was used with an ECG gating system. The CMR scans were performed using a previously published protocol [56]. To evaluate the potential for fibrosis, myocardial T1 mapping technique was applied using a protocol established from an unpublished GRMD cardiac study. The modified Look-Locker inversion recovery (MOLLI) sequence was used to obtain a pre-contrast myocardial T1 map followed by 0.2 mmol/kg gadolinium intravenous injection. A post-contrast myocardial T1 map was obtained 10 mins after gadolinium injection using the MOLLI sequence. All CMR images were imported to a Siemens Syngo workstation (Siemens Medical Solutions) and Siemens Argus software (Siemens Medical Solutions) was used for analysis. The pre- (native T1) and post-contrast T1 relaxation times of the LV anterolateral to inferior wall were measured and further used for extracellular volume (ECV) calculation [60, 61]. Blood was collected immediately after the CMR scan to determine the hematocrit value. The ECV was calculated using the formula: *ECV = (1-hematocrit) x [(1/post contrast T1 myo– 1/native T1 myo) / (1/post contrast T1 blood– 1/native T1 blood)]*. ECV was expressed as a percentage.

### Histology and assessment of histopathology

Multiple muscle and visceral organs were sampled at necropsy. For pathological analysis, muscle samples were taken by biopsy or at necropsy from GRMD, immediately frozen in isopentane (2-methylbutane) cooled in liquid nitrogen, and stored at -80°C. Samples were shipped to Nationwide Children’s Hospital where they were stored at -80°C. At processing for histopathologic evaluation, samples were transferred to liquid nitrogen cooled SUVA34A (Freon analogue). Frozen skeletal muscle blocks were cross sectioned from the midsection of the muscle at 10μm thickness on a cryostat.

Some muscle biopsies and all non-muscle organs were also fixed in 10% neutral-buffered formalin and shipped to the Biopathology Core at Nationwide Children’s Hospital, which performed routine processing, sectioning, and mounting of thin sections on slides, followed by H&E staining. Tissues were embedded in paraffin, cut, washed in tap water, stained in Gill’s 3 Hematoxylin (Fisher Scientific; Pittsburgh, PA) for 2 minutes, washed in tap water, then bluing agent, then washed again in tap water. Sections were then stained in Eosin (Fisher Scientific; Pittsburgh, PA) for 1 minute and washed 3 times in clean 100% ethanol. The sections were cleared in xylene and mounted in the xylene-based mounting media Cytoseal.

Fibrosis index was measured in formalin-fixed slides stained with H&E taken from biopsies of diaphragm, triceps (lateral head), deep pectoral, biceps brachii, biceps femoris, cranial sartorius, cranial tibialis, middle gluteal, rectus femoris, long digital extensor, gastrocnemius, semimembranosus, semitendinosus, and vastus lateralis muscle. The same muscles were compared between all cohorts, but in a few instances, muscle sections were not analyzed due to poor section quality or because the muscle was not biopsied for a particular animal. Fibrosis index scores were assessed in a blinded manner as follows, taking all tissue on the section into account: 0- no fibrosis, 1-minimal fibrosis (0% to 5% non-muscle area), 2-mild fibrosis (5% and 15% non-muscle area), 3-moderate fibrosis (15% and 25% non-muscle area), 4-severe fibrosis (25% and 50% non-muscle area), and 5 very severe fibrosis (50% non-muscle area).

### Immunostaining and lectin staining

For immunostaining, 10μm cryostat cut frozen muscle sections were blocked in 5% goat serum in PBS at room temperature for 1 hour. Sections were then incubated with anti-Laminin (Sigma Aldrich; St. Louis, MO) at 1μg/ml, at room temperature for 2 hours, or with one of the following antibodies: anti-Utrophin (Novocastra, Buffalo Grove, IL) at 7.5 μg/ml, anti-Plectin 1 (Santa Cruz, Dallas, TX) at 1 μg/ml, anti-Laminin α5 (Abcam, Cambridge, MA) at 10 μg/ml, anti-α-dystroglycan (clone IIH6C4, Sigma Aldrich, St. Louis, MO) at 1:100, or anti-dystroglycan (R&D Systems, Minneapolis, MN) at 5 μg/ml at 4° C overnight. Sections were incubated in appropriate Cy3-conjugated secondary antibodies (Jackson Immunoresearch; West Grove, PA) at 7.5μg/mL and FITC-conjugated *Wisteria floribunda* agglutinin (WFA, Vector Laboratories, Burlingame, CA) at 1μg/ml for 1 hour at room temperature and then mounted using ProLong Gold Antifade Mountant (Invitrogen, Carlsbad, CA).

### Lectin precipitations and western blotting

Frozen muscle blocks were sectioned on a cryostat so that approximately 12mg was collected from each sample. Muscle was digested in Tris-buffered saline (TBS; 25 mM Tris, 150 mM NaCl, pH 7.5) containing 1% NP-40, 0.5 mM EDTA, and cOmplete protease inhibitors (Roche; Indianapolis, IN, diluted according to manufacturer’s instructions) overnight. Protein was quantitated using a micro-BCA protein assay (Thermo Scientific; Waltham, MA). Agarose-bound *Wisteria Floribunda* Agglutinin (WFA, Vector Laboratories, Burlingame, CA) or *Triticum Vulgare* (Wheat Germ) Agglutinin (WGA, EY Laboratories, San Mateo, CA) was prepared by washing in lysis buffer 3 times and resuspended in lysis buffer. Extracted protein (150 μg, 500 μg, 1 mg, or 3 mg) was incubated with WFA agarose or WGA agarose overnight at 4° C on a rocker. The beads were washed 3 times with low salt TBST (50mM Tris-HCl, pH 7.4, 100mM NaCl, 0.05% Tween-20). After the last wash, the samples were resuspended in NuPage LDS sample buffer (Invitrogen, Carlsbad, CA) with β-mercaptoethanol. Samples were boiled for 10 minutes, then separated on a Bolt 4–12% Bis-Tris Plus gel (Life Technologies; Grand Island, NY) and transferred to nitrocellulose. The membrane was blocked in 5% nonfat dry milk in low salt TBST then probed with anti-α-dystroglycan antibody, clone IIH6C4 (Millipore, Burlington, MA) or with a polyclonal antibody made to the dystroglycan polypeptide. After washing, horseradish peroxidase-coupled secondary antibody was added and blots were developed using the ECL chemiluminescence method (Lumigen; Southfield, MI) after further washing.

For immunoblots of whole cell protein lysates, frozen muscle blocks were sectioned on a cryostat. Muscle was digested in Tris-buffered saline (TBS; 25 mM Tris, 150 mM NaCl, pH 7.5) containing 1% NP-40, 0.5 mM EDTA, and cOmplete protease inhibitors (Roche; Indianapolis, IN, diluted according to manufacturer’s instructions) overnight. Protein was quantitated using a micro-BCA protein assay (Thermo Scientific; Waltham, MA). 40 μg of protein was diluted in NuPage LDS sample buffer (Invitrogen, Carlsbad, CA) with 0.1M β-mercaptoethanol. Samples were boiled for 10 minutes, then separated on a Bolt 4–12% Bis-Tris Plus gel (Life Technologies; Grand Island, NY), transferred to nitrocellulose, and immunoblotted as above using anti-Dystrophin (DYS1, Leica Biosystems, Buffalo Grove, IL), anti-Utrophin (DRP2, Leica Biosystems, Buffalo Grove, IL) or anti-glyceraldehyde 3-phosphate dehydrogenase (GAPDH, Sigma Aldrich, St. Louis, MO) as a loading/transfer control.

### Enzyme-linked immunosorbent assay (ELISA)

Recombinant (r) AAV2 and AAV8 were obtained from SignaGen (Rockville, MD). All other rAAV viral vectors were obtained from the Viral Vector Core facility at Nationwide Children’s Hospital, where they were produced by the triple transfection method in HEK293 cells and highly purified using density centrifugation and anion exchange chromatography. The identity of various serotypes was confirmed using serotype-specific monoclonal antibodies, as before [62]. Plates were coated overnight at 4° C in coating solution with or without rAAV particles (0.2M bicarbonate buffer, pH 9.4 with or without 2x10^9^ viral particles/well of rAAVrh74, rAAV1, rAAV2, rAAV6, rAAV8, or rAAV9). Plates were blocked for 1 hour at 37° C in blocking buffer (5% milk, 1% goat serum in PBS). Initial screening of samples was done by diluting the serum 1:50 in blocking buffer and adding 100μl to each of 4 wells. Each sample was assessed in duplicate in wells coated with viral particles and with bicarbonate buffer alone to adjust for background. Samples were incubated at 37°C for one hour and then each plate was washed 5 times with wash buffer (PBS with 0.05% Tween-20). HRP-conjugated secondary antibody was then added at a 1:10,000 dilution (Dog IgG heavy and light chain antibody, Bethyl Laboratories, Montgomery, TX), in blocking buffer and incubated at room temperature for 30 minutes in the dark. Each plate was washed 5 times again with wash buffer. HRP substrate reagent (R&D Systems, Minneapolis, MN) was added to each well and allowed to develop in the dark for 15 minutes. The reaction was stopped with 1N sulfuric acid and the optical density of each well was read on a Synergy2 Plate Reader (BioTek, Winooski, VT) at 450 nm.

Any samples that tested positive at 1:50 were retested by ELISA with a dilution series from 1:100 using subsequent 1:2 dilutions until a dilution was identified where at least a two-fold increase in signal was no longer obtained. For each sample, the mean optical density (OD) was calculated by subtracting the background value (-viral particle wells) from the sample value (+viral particle wells) and determining the signal/background ratio. The serum sample was called AAV-positive if the signal/background ratio was greater than 2. Background signals remained constant throughout at an OD of 0.1 to 0.2, making for little or no variability in the quotient used to determine 2-fold or greater differences. For the GALGT2 (B4GALNT2) ELISA, the same procedure was followed except wells were coated using 100 ng of human GALGT2 protein (Abnova, Taipei, Taiwan).

### Gamma interferon enzyme-linked immunospot (ELISpot) assay

Blood samples were collected at pre-injection, 45 days post-injection, and 90 days post-injection. Isolated cells were counted, and a 10^6^ splenocyte/mL solution was used to seed wells of a 96-well PVDF-coated filter plate (Millipore, Burlington, MA) with 2x10^5^ cells/well. Three pools of overlapping peptides comprising the entire sequence of the rAAVrh74 capsid protein, or two pools of peptides comprising the entire sequence of the human GALGT2 protein, all 18 amino acids in length, were added at 0.2μg to cells to assess T cell responses. Although use of the human GALGT2 peptide library was not perfectly matched to the canine GALGT2 sequence (82.8% identical), the human GALGT2 protein sequence was used for these assays. Two μg of Concanavalin A (ConA) was used as a positive control, and vehicle alone (DMSO) was used as a negative control. All assays were performed in duplicate. After 36–48 hours incubation in a sterile cell incubator at 37°C with 5% CO_2_, cells were washed and developed for canine γ-interferon (R&D Systems, Minneapolis, MN). Positive cells were counted on a plate reader as previously described [63, 64].

### Quantitative polymerase chain reaction (qPCR)

Qiagen DNeasy Blood and Tissue kit (Qiagen, Germantown, MD) was used to extract genomic DNA from frozen muscle and tissue following the manufacturer’s protocol. 100ng of genomic DNA was assayed by qPCR using primers specific to the AAV vector genome: Forward 5’- CCT CAG TGG ATG TTG CCT TTA -3’, Probe 5’-/56-FAM/AAA GCT GCG/ZEN/GAA TTG TAC CCG C/3IABkFQ/-3’ and Reverse 5’- ATG CCA AGT CCT AAG ACT AAA AC -3’. The pAAV.MHCK7.*GALGT2*(canine) plasmid was cut with SwaI and purified for use as a linear DNA standard. This standard was measured between 50 and 5 million copies in logarithmic increments to generate a linear calibration, for which the Pearson correlation coefficient always equaled or exceeded 0.98. qPCR measures were performed on a TaqMan ABI 7500 sequence detection system (Applied Biosystems, Foster City, CA). Each sample was run in triplicate and the values were averaged. Some samples were spiked with 100 copies of standard and measured to insure no quenching of signal by genomic DNA or by contaminants from the extraction procedure.

To calculate vg/nucleus, the number of vector genomes per μg of genomic canine DNA was divided by 1.8x10^5^ vg/nucleus, assuming 5.4 pg of genomic DNA per diploid nucleus.

### Semi-quantitative real time polymerase chain reaction (qRT-PCR)

Total RNA was isolated using Trizol reagent (Invitrogen, Carlsbad CA) from frozen blocks of cranial sartorius muscle or from heart. RNA was purified using Direct-zol RNA minipreps (Zymo Research, Irvine, CA). Relative transcription levels were assessed by qRT-PCR using the 2-(Delta Delta C(T)) method with 18S rRNA as an internal reference [65]. A high capacity cDNA archive kit (Applied Biosystems, Foster City, CA) was used to reverse transcribe RNA per manufacturer guidelines. Samples were subjected to real-time PCR in duplicate using a TaqMan ABI 7500 sequence detection system (Applied Biosystems, Foster City, CA). Premade primer/probe sets for Integrin β1 (Itgb1, qCfaCIP0003159), Utrophin (qCfaCIP0016479), DMD (qCfaCIP0019340), and Laminin α4 (qCfaCIP0001441) were purchased from Bio-Rad Laboratories (Hercules, CA). Other primer/probe sets were designed using Primer3 (primer3.ut.ee) and synthesized (Integrated DNA Technologies, Coralville, IA) to recognize canine Agrin (forward: 5’-GCATCCGCCTACTCAGTC-3’, probe: 5’-/56-FAM/CAATGTGAC/ZEN/ATGCAGCTTCGGCAG/3IABkFQ/-3’, reverse: 5’- CAGGCATGTGGCTGTCT-3’), canine Plectin 1 (forward: 5’- GTGCAGACCCTCAAGGATG-3’, probe: 5’-/56-FAM/ CAGATGTAC/ZEN/CGCAGAGTGTACCGC/3IABkFQ/-3’, reverse: 5’- CTTTAGCCGGAGGTTGTACTC-3’), canine Laminin α-2 (forward: 5’- GGGTTGCCCTACTTCAGTTAC-3’, probe: 5’-56-FAM/ ACCAAGATC/ZEN/AACGACGGCCAGT/3IABkFQ/-3’, reverse: 5’-CCTTCTTGCTTAATTCGCATAATCT-3’), canine Laminin α-5 (forward: 5’-CTTCCTGAGCCACGGAT-3’, probe: 5’-56-FAM/TTTGTCGCG/ZEN/CAGACAGAAGGC/3IABkFQ/-3’, reverse: 5’-CTTCTCCCACCGCACAG-3’), canine GALGT2 (forward: 5’-TTGAAGTGCTGGTGGATGTC-3’, probe: 5’-/56-FAM/AACTGGATG/ZEN/TGGTAGGTGGCAGTG/3IABkFQ/-3’, reverse: 5’-GCAACAGCTTAAACTGGAACAC-3’), canine DAG1 (forward: 5’-AGTGACCATTCCCATGGATTTA-3’, probe: 5’-56-FAM/AACTCGTCA/ZEN/AGGTGTCAGCAGTGG/3IABkFQ/-3’, reverse: 5’-TATCGGTGTCAAGAGGGAGA-3’), canine Integrin α-7 (forward: 5’-ACATCTGGCTTTGGCTACTC-3’, probe: 5’-56-FAM/CCGATCTCA/ZEN/ACAACGATGGCTGGA/3IABkFQ/-3’, reverse: 5’-TCTTGGCGCTCAAAGAAGT-3’). Relative mRNA levels were averaged and compared to untreated GRMD samples.

### Serum chemistries and hematology measures

Blood was collected pre-treatment, at 24 and 72 hours, and on days 45 and 90, with subsequent analysis of hematologic and serum chemistry tests by the Texas A&M clinical chemistry core facility. Serum chemistry measures included: Total (Serum) Protein, Albumin, Calcium, Phosphorus, Glucose, Blood Urea Nitrogen, Creatinine, Total Bilirubin, Alkaline phosphatase, Creatine Kinase, Aspartate Aminotransferase, Alanine Aminotransferase, Globulin, Albumin to Globulin ratio, Gamma Glutamyl Transferase, Glutamate Dehydrogenase, Sodium, Potassium, Sodium to Potassium ratio, and Chloride. Hematology measures included: White Blood Cells (Leukocyte), Neutrophils, Lymphocytes, Monocytes, Eosinophils, Red Blood Cell (Erythrocyte), Hemoglobin, Hematocrit, Mean Corpuscular Volume, Mean Corpuscular Hemoglobin, Mean Corpuscular Hemoglobin Concentration, Red Blood Cell Distribution Width, Platelet Count, and Procalcitonin.

### Statistical analysis

For statistical analysis of pathology measures, the Mann-Whitney test was used to compare between any two groups. P < 0.05 was considered significant in this study. All other comparisons between two groups were done using a two-tailed unpaired Student’s t test. Data were analyzed using Prism 7.0e software (GraphPad, San Diego CA).

## Results

### Muscle biodistribution, gene expression, and glycosylation after intravenous treatment with rAAVrh74.MHCK7.GALGT2

The standard window for assessing GRMD dogs in preclinical studies is at 3–6 months of age, when signs of disease progress markedly, corresponding to 5–10 years in DMD [19, 21]. Accordingly, at 3 months, five GRMD dogs were given a single intravenous (IV) dose of rAAVrh74.MHCK7.*GALGT2*, with four receiving 2x10^14^vg/kg and one (Nani) 6x10^14^vg/kg, while one GRMD dog was not treated with AAV. An additional three untreated GRMD dogs that had been assessed blindly in a separate preclinical study using the same experimental design, about 2 years previously, were also included, creating a control group of four GRMD dogs for comparisons. Dogs were assessed with a standard group of functional studies at 3 months (baseline) before dosing and reassessed at 4½ and 6 months of age, corresponding to 1.5 and 3 months after treatment. In addition to comparisons to the control group, functional data from treated GRMD dogs were compared to historical measures from untreated GRMD and wild type littermate golden retriever (GR) dogs [66]. Because this study entailed a 3-month treatment period and single-stranded AAV.*GALGT2* vectors typically take 3–4 weeks after muscle transduction to achieve full gene expression [36], we chose to use the stronger MHCK7 [67, 68] promoter instead of the MCK (CK7-like [69]) promoter, which is currently being tested in a phase 1/2a DMD clinical trial using rAAVrh74.MCK.*GALGT2* (NCT03333590).

**Fig 1:**
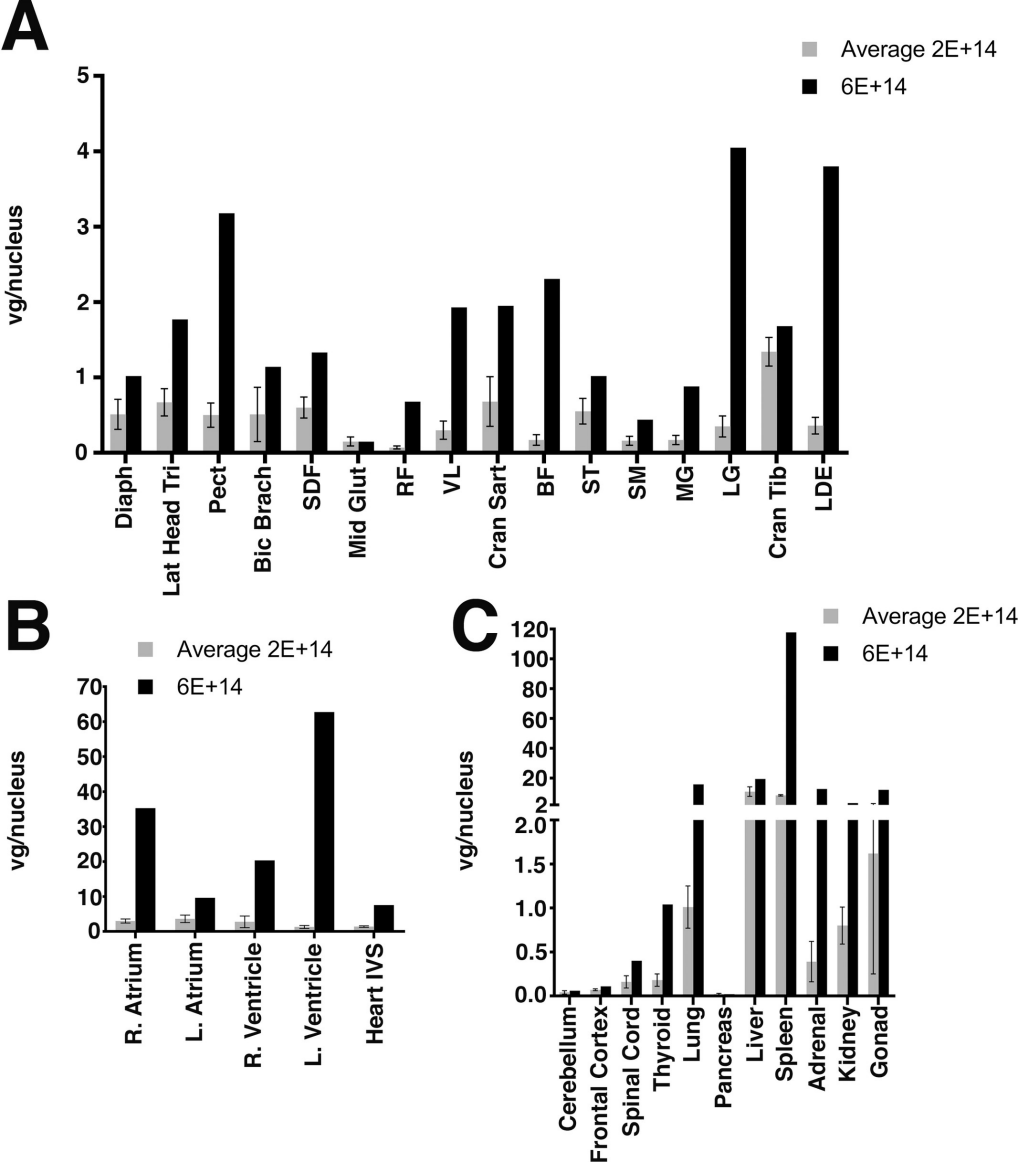
Biodistribution of AAV genomes in muscles and non-muscle organs after rAAVrh74.MHCK7.*GALGT2* treatment of GRMD dogs. rAAV vector genomes were measured in skeletal muscles (A), heart (B), and non-muscle organs (C) by qPCR. Four GRMD dogs were dosed with 2x10^14^vg/kg (2E+14), with average transduction levels shown (lighter bars), while one GRMD dog was dosed with 6x10^14^vg/kg (black bars, 6E+14). Abbreviations: Diaph (Diaphragm), Lat (Lateral) Head Tri (Triceps), Pect (Deep Pectoral), Bic Brach (Biceps Brachii), SDF (Superficial Digital Flexor), Mid Glut (Middle Gluteal), RF (Rectus Femoris), VL (Vastus Lateralis), Cran Sart (Cranial Sartorius), BF (Biceps Femoris), ST (Semitendinosus), SM (Semimembranosus), MG (Medial head, Gastrocnemius), LG (Lateral head, Gastrocnemius), Cran Tib (Cranial Tibialis), LDE (Long Digital Extensor), R (Right), L (Left), IVS (Interventricular Septum). Errors are SD for n = 4 for 2x10^14^vg/kg group.

We first analyzed AAV biodistribution in skeletal muscles, heart, and non-muscle organs taken at necropsy at 6-months of age, corresponding to 3 months after gene therapy treatment (Fig 1). Skeletal muscles assessed included diaphragm, triceps (lateral head), deep pectoral, biceps brachii, biceps femoris, cranial sartorius, cranial tibialis, middle gluteal, rectus femoris, long digital extensor, gastrocnemius (lateral and medial heads), superficial digital flexor, semimembranosus, semitendinosus, and vastus lateralis (Fig 1A). Cardiac samples were taken from the right and left atrium, right and left ventricle, and the interventricular septum (Fig 1B). At the 2x10^14^vg/kg IV dose of rAAVrh74.MHCK7.*GALGT2*, all regions of the heart were transduced with between 1 and 3 vector genomes (vg) per nucleus. Skeletal muscles, by contrast, were not as well-transduced at this dose; most muscles had 0.25 to 0.5vg/nucleus (or between 1 in every 4 or 2 nuclei transduced, respectively). The only muscle transduced with >1vg/nucleus was the cranial tibialis. As with our previous studies in rhesus macaques [69], the muscle-to-muscle variability between GRMD dogs given the same dose of AAV was significant (S1 Fig). Several muscles, including the middle gluteal, rectus femoris, and semimembranosus, showed low average levels of transduction, with about only 1 in 10 nuclei transduced (or 0.1vg/nucleus). The one dog given the 6x10^14^vg/kg dose had higher levels of vg transduction in most heart and skeletal muscles studied, with all five heart regions sampled having >7 vg/nucleus and almost all skeletal muscles transduced with >1vg/nucleus. Again, there were a few exceptions, including the middle gluteal, rectus femoris, and semimembranosus, which were transduced poorly even at this higher dose.

Non-muscle organs assessed included the brain (cerebellum and frontal cortex), spinal cord, lung, pancreas, liver, spleen, adrenal gland, kidney, and gonads (ovary or testicle) (Fig 1C). As expected, based on previous work in mice and rhesus macaques [68, 69], the greatest transduction for rAAVrh74 was in the liver and spleen (12 and 8 vg/nucleus, respectively), while the lowest level of transduction was in the brain (both cerebellum and frontal cortex <0.1vg/nucleus). Unexpectedly, pancreas also showed a very low level of transduction (also <0.1vg/nucleus), a finding not seen previously in mice or in macaques [68, 69]. Transduction of the spinal cord was significantly higher than brain, again as before [68, 69]. The higher 6x10^14^vg/kg dose yielded greater levels of transduction in almost all non-muscle organs than was found at the lower 2x10^14^vg/kg dose.

We next assessed the fold-change in *GALGT2* gene expression by qRT-PCR, using endogenous canine *GALGT2* in archived muscle from untreated GRMD and normal GR dogs as the comparative standard and 18S ribosomal RNA gene as the internal standard. The fold-change in *GALGT2* gene expression was measured in skeletal muscle (cranial sartorius, CS) and heart (left ventricle, HLV). *GALGT2* gene expression did not greatly differ (no more than two-fold, not shown) between untreated GRMD and otherwise normal GR dogs. GRMD dogs dosed with 2x10^14^vg/kg of rAAVrh74.MHCK7.*GALGT2* averaged a 102±33-fold increase in *GALGT2* gene expression in CS and an 82±26-fold increase in HLV relative to untreated GRMD dogs. The relatively high fold-increase in CS skeletal muscle relative to heart, compared to the lower vg biodistribution data (Fig 1), was caused by increased endogenous baseline canine *GALGT2* expression in heart relative to CS (not shown). Interestingly, the one GRMD dog given the higher (6x10^14^vg/kg) AAV dose had similar levels, showing a 99-fold increase in CS and a 128-fold increase in HLV. While this level of increase was similar to that seen for the low dose cohort, this may reflect the variability commonly seen between different muscle samples [69].

**Fig 2:**
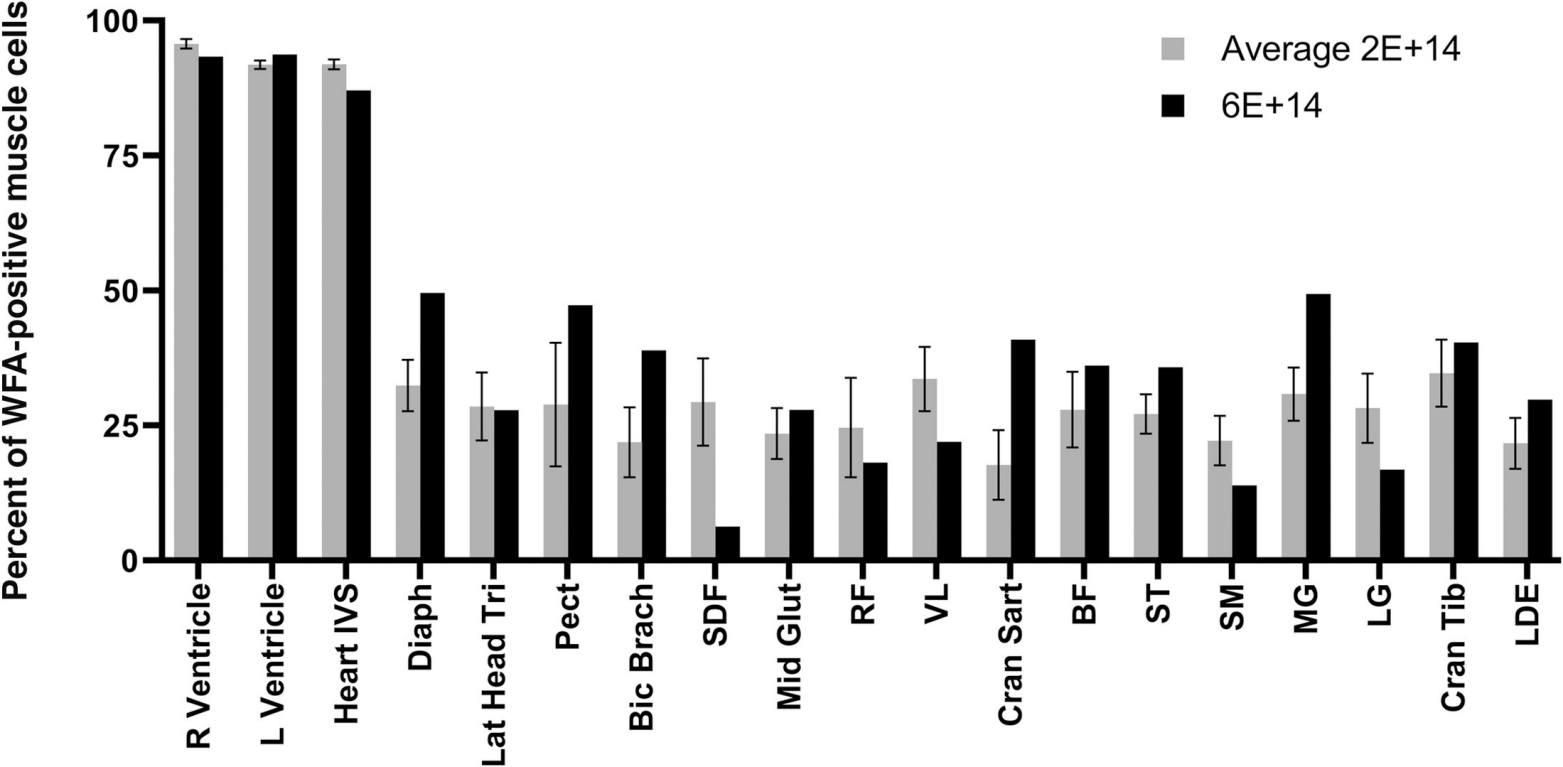
*GALGT2*-induced glycosylation in heart and skeletal muscles after rAAVrh74.MHCK7.*GALGT2* treatment of GRMD dogs. WFA staining was scored in each cardiomyocyte or skeletal myofiber in muscle sections taken from four GRMD dogs dosed with 2x10^14^vg/kg (lighter bars 2E+14) or one GRMD dog with 6x10^14^vg/kg (black bars, 6E+14). Errors are SD for n = 4 for the 2x10^14^vg/kg group.

**Fig 3:**
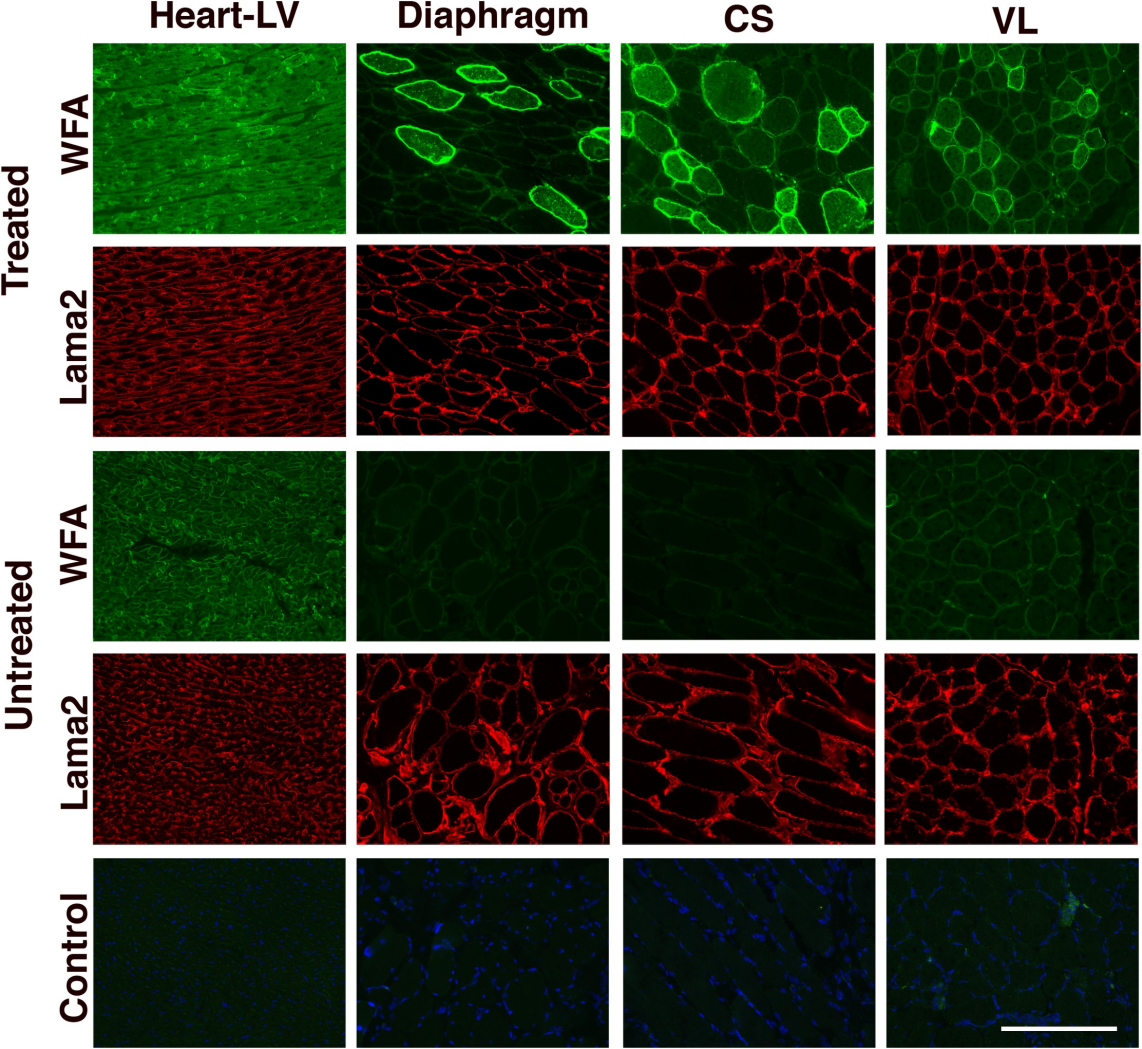
WFA staining after treatment of GRMD dogs with rAAVh74.MHCK7.*GALGT2*. Muscle sections from 6-month-old GRMD dogs treated (or untreated) at 3 months of age with 2x10^14^vg/kg of rAAVrh74.MHCK7.*GALGT2* were assayed for *GALGT2*-induced glycosylation. Sections were stained with WFA (green), to identify βGalNAc made by GALGT2, and with antibody to laminin α2 (Lama2, red), to identify sarcolemmal membranes. Control shows merged red, green and blue fluorescence signal without WFA and with only secondary antibody, with DAPI (blue) added as a nuclear co-stain. Sections were stained from heart (left ventricle, LV), diaphragm, cranial sartorius (CS), and vastus lateralis (VL). All exposures are time matched. Bar is 100μm for all panels.

We next assessed *GALGT2*-induced glycosylation in heart and skeletal muscles (Figs 2 and 3). Glycosylation was assessed by staining with the βGalNAc-binding lectin *Wisteria Floribunda* Agglutinin (or WFA). Wild type GR muscles showed βGalNAc sarcolemmal staining concentrated primarily at NMJs (not shown), as previously reported [70], with little or no extrasynaptic muscle fiber sarcolemmal staining. Low level WFA staining was increased in some muscles from untreated GRMD dogs, much as can be seen in certain *mdx* mouse skeletal muscles compared to wild type [17], but the intensity of this low-level WFA staining in untreated GRMD muscles was dramatically different than positively stained fibers in AAV.*GALGT2*-treated muscles (Fig 3). Notably, levels of WFA staining in treated muscles varied markedly across specific muscles of individual dogs (S2 Fig). For the 6x10^14^vg/kg dose, the diaphragm, deep pectoral, and medial head of the gastrocnemius showed about 50% of skeletal myofibers with increased WFA staining, comparable or higher than levels seen with the lower dose. The majority of skeletal muscles studied, however, did not show a clear increase in glycosylation at the higher dose, suggesting either a kinetic hindrance to achieving higher glycosylation or a saturation of glycosylation at the lower dose.

WFA staining in heart was elevated in untreated GRMD compared to normal GR and was further increased after treatment with rAAVrh74.MHCK7.*GALGT2* (Fig 3). At the 2x10^14^vg/kg dose of rAAVrh74.MHCK7.*GALGT2*, greater than 90% of all cardiomyocytes in the right and left ventricle and the interventricular septum showed increased WFA staining intensity. Glycosylation in regions of the heart in the one dog given the 6x10^14^vg/kg dose of rAAVrh74.MHCK7.*GALGT2* was similarly high.

### Assessment of skeletal and cardiac muscle function

This was an unblinded study, so we knew that dogs in the treatment group were receiving *GALGT2*, introducing an inherent bias in data collection and interpretation. On the other hand, the control group was blinded to treatment. Further, with regard to data interpretation, because phenotypic variation in GRMD and the associated large standard deviation makes it difficult to achieve significance subsequent to treatment, especially with the small groups used, our experimental design typically assessed each dog as its own control, determining the % change from baseline at 3 months to the interim and final end points at 4½ and 6 months.

Body mass obviously influences strength and must be considered as an additional variable. For this reason, we usually assess both absolute and body-mass-corrected force/torque, again determining % change at the same time points (Table 1). Notably, GRMD dogs are smaller than their wild type littermates, allowing body mass to be used as a separate outcome parameter, with increases in treated dogs suggesting potential efficacy. In assessing body mass, the GRMD 2x10^14^vg/kg *GALGT2* treatment group was composed of relatively small dogs, whose baseline body mass (4.65±1.32 kg) was lower than that of the four controls (6.08±0.82 kg), although the difference wasn’t significant (p = 0.2). In tracking body mass at subsequent time points, the treated dogs showed a greater % change from baseline when compared to the control group at both 4½ (78.7±19.1% versus 60.8±26.8%) and 6 (142.2±44.5% versus 85.4±25.7%) months of age. However, again, neither value reached significance (p = 0.46 and 0.49).

**Table 1:** Functional data from GRMD dogs.

Day/Age	Body Mass	Mass-Corrected Tetanic Torque (Nm/kg)		ECD (1–30) (%)	TTJ Angle (^o^)	Pelvic Angle (^o^)	6MWT Height Adjusted (m/cm)
		Extension*	Flexion				
*GALGT2*-Treated Dogs (2x10^14^vg/kg)							
0 (3 mos)	4.65±1.32	0.11±0.02	0.06±0.00	46.2±8.44	153.5±14.5	39.0±2.92	9.90±2.94
45 (4½ mos)	6.18±3.86	0.17±0.06	0.07±0.00	48.6±20.5	142.5±20.0	45.3±4.0	
% Change (0–45)	78.7±19.1	59.5±57.8	14.2±16.4	7.38±14.3	(-6.8)±8.22	16.1±5.87	
90 (6 mos)	10.7±1.14	0.19±0.09	0.07±0.01	45.0±24.3	131.0±16.2	42.3±6.18	5.40±2.20
% Change (0–90)	142.2±44.5	79.0±82	26.1±25.4	(-7.71)±41.2	(-10.6)±11.3	14.7±6.33	(-11.8)±39.4
*GALGT2*-Treated Dog (6x10^14^vg/kg)							
0 (3 mos)	3.6	0.24	0.06	50.0	156	40	
45 (4½ mos)	6.8	0.17	0.07	72.0	153	42	
% Change (0–45)	88.9	(-28.3)	21.4	44.0	(-1.92)		5
90 (6 mos)	8.6	0.22	0.05	40	150	38	
% Change (0–90)	138.9	(-8.9)	(-12.5)	(-20)	(-3.85)		(-5)
Control Dogs							
0 (3 mos)	6.08±0.82	0.22±0.05	0.08±0.01	52.1±22.7	158.5±5.03	36.5±2.29	6.07±2.00
45 (4½ mos)	9.6±1.03	0.22±0.04	0.08±0.03	64.1±22.0	149.3±7.86	52.5±3.20	
% Change (0–45)	60.8±26.8	9.7±45.6	3.95±40.3	39.9±62.6	(-5.85)±3.79	44.3±11.2	
90 (6 mos)	11.2 ±1.61	0.26±0.07	0.09±0.02	54.8±13.9	153.0±12.3	45.5±5.32	5.68±0.68
% Change (0–90)	85.4±25.7	28.9±61.8	7.93±28.9	26.0±54.2	(-3.42)±3.51	25.6±20.8	6.60±34.4
Natural History GRMD Controls^#^							
0 (3 mos)	7.36±1.29	0.20±0.05	0.06±0.02	36.5±11.5	159.4±3.72	NA^$^	7.3
90 (6 mos)	13.4±2.20	0.18±0.10	0.08±0.02	54.3±14.7	151.3±10.2	48.3±5.26	4.5
% Change (0–90)^^^	84.6±31.2	-6.68±40.1	22.0±36.4	69.84±95.7	(-5.09)±5.62	NA	-38.4
Natural History Normal Controls^#^							
0 (3 mos)	8.46±0.63	0.30±0.04	0.13±0.01	26.80 ± 5.92	167.9±3.94	43.8±5.12^$^	8.0
90 (6 mos)	17.8±0.44	0.36±0.07	0.18±0.02	17.86 ± 5.20	160.9±3.40	36.1±5.98	8.6
% Change (0–90)^^^	111.5±17.2	19.3±12.1	42.6±13.7	-21.99±29.0	(-4.15)±1.82	(-6.09)±12.4	7.5

*Removing outliers Tony and Asiago from the control and GALGT2-treated data sets such that each group has an n = 3: CONTROLS: Baseline = 0.243±0.025; Interim = 0.203±0.029; Final = 0.230±0.049; % Change Baseline-Interim = (-16.0)±11.6; Baseline-Final = (-5.93)±15.6. GALGT2-TREATED: Baseline = 0.11±0.022; Interim = 0.137±0.012; Final = 0.147±0.056; % Change Baseline-Interim = 27.8±20.8; Baseline-Final = 35.5±37.6.

^#^Mean±SD* control values for all but 6MWT are from Kornegay JN, et al: Skelet Muscle 4:18, 2014; Controls for 6MWT are median values and are from Acosta AR, et al: Neuromuscul Disord 26:865–72, 2016.

^^^Mean % Change was calculated based on the changes in individual dog values versus the change in the overall mean values.

^$^There were 3 month values for only 1 GRMD and 4 Normal dogs; thus, % change was not calculated for GRMD and represented only four normal dogs.

For assessment of skeletal muscle function, we measured body-mass-corrected tibiotarsal joint (TTJ) extension and flexion torque, eccentric contraction decrement in TTJ flexion torque after 30 repeated contractions, TTJ and pelvic joint angles, and height-adjusted 6-minute walk test (6MWT) (Table 1). Values from the four GRMD dogs treated with 2x10^14^vg/kg rAAVrh74.MHCK7.*GALGT2* and the one dog (Nani) given the higher 6x10^14^vg/kg dose were compared to both the control group and historical published data from untreated GRMD and GR dogs [66]. Although firm conclusions were limited by the low number of dogs studied and by the variability in absolute measures from dog to dog (S1 Table), the low dose cohort of four dogs showed a trend toward improvement in all but TTJ angle (Table 1). Note that the percentage change calculations here were done by averaging the percentage change for each individual dog between the 2 time points and do not represent the percentage change between the average values for those measures.

Our GRMD natural history studies have shown that body-mass extension torque/force increases minimally or even declines between 3 and 6 months of age, whereas flexion tends to increase [52]. Accordingly, we have used extension torque/force as the primary outcome parameter in GRMD preclinical trials (Table 1). The 2x10^14^vg/kg *GALGT2* treatment group had significantly lower baseline body-mass-corrected extension torque (0.11±0.02) compared to untreated GRMD controls (0.22±0.05) (p < 0.05). Values no longer differed at 4½ (0.17±0.06 versus 0.22±0.04; p = 0.23) or 6 (0.19±0.09 versus 0.26±0.07; p = 0.4) months. These increases were reflected by higher values for % change at 4½ (59.5±57.8 versus 9.7±45.6%; p = 0.2) and 6 (79.0±82 vs. 28.9±61.8%; p = 0.34) months and also compared favorably to the 6.68±40.08% decline seen in the natural history GRMD dogs [66]. Each of the 2x10^14^vg/kg *GALGT2* treatment and control groups had an outlier (Tony in the controls and Asiago in the treatment group). If these two dogs were removed and the remaining three dogs in each group were compared, the difference persisted (treatment, 35.5±37.6%; controls, -5.93±15.6%; p = 0.1).

A less pronounced effect was seen on assessment of flexion torque (Table 1). The 2x10^14^vg/kg *GALGT2* treatment group, again, had lower baseline values (0.06±0.00 vs. 0.08±0.01; p < 0.05), with the difference no longer being significant at 4½ and 6 months. This was reflected in higher % change values, as the treated GRMD dogs showed a 26.1±25.4% increase from 3 to 6 months versus only a 7.93±28.9% increase in controls (p = 0.34). Notably, in the natural history study, flexion torque increased 22.0±36.4%, comparable to the level seen in *GALGT2*-treated dogs, diminishing the likelihood that this was a treatment effect.

Values for ECD should, in principle, be particularly sensitive to treatments such as *GALGT2* intended to stabilize the sarcolemma. Based on natural history studies [54], the degree of ECD increases from 3–6 months in GRMD (Table 1), presumably because of continued membrane destabilization, whereas ECD of wild type dogs lessens, likely reflecting further membrane maturity (Table 1). This expected increase in ECD from 3 to 6 months was evident in the GRMD controls, with a mean increase of 26.0±54.2%, whereas the *GALGT2* treatment group showed a mean decrease of 7.71±41.2%, in principle reflecting greater membrane stability. Nonetheless, the difference was not significant (p = 0.49), even when outliers were removed (p = 0.4).

Postural changes in GRMD, demonstrated through measurement of joint angles, can distinguish treatment effects [21]. We have focused on changes in the TTJ and pelvic angles, associated with the plantigrade stance and pelvic tilt typical of GRMD, respectively. The % change in TTJ did not differ between *GALGT2*-treated (-10.6±11.3%) and control dogs (-3.42±3.51%), with both values comparable to the natural history value of -5.09±5.62%. The increase in pelvic angle typically seen in GRMD at 6 months showed a less pronounced, but insignificant, increase from 3–6 months in treated (14.7±6.33%) versus control (25.6±20.8%) GRMD dogs. Finally, assessment of 6MWT was limited because data at both 3 and 6 months was only available from a total of five dogs, with the three controls showing a 6.60±34.4% increase in distance walked compared to a 11.8±39.4% decrease in the two treated dogs.

Potentially reflecting variable levels of *GALGT2* translation but also in keeping with inherent phenotypic variation, functional values differed considerably across treated GRMD dogs and untreated controls. As an example, extension torque in Asiago (0.328 Nm/kg) at 6 months approached natural history normal values (0.36±0.07 mN/kg for GRs) (S1 Table). Similarly, the degree of ECD was less pronounced in two of the treated GRMD dogs (Destiny and Ash) at 6 months, in line with normal natural history controls. These differences amongst individual dogs could provide insight into *GALGT2*’s functional effects. Tibiotarsal joint extension is achieved through contraction by muscles of the caudal tibial compartment, namely the medial and lateral heads of the gastrocnemius muscle and the superficial digital flexor. Flexion is accomplished by cranial tibial compartment muscles, chiefly the cranial tibialis. Thus, disproportionate increases in extension torque or ECD could be driven by analogous greater levels of glycosylation of caudal and cranial tibial compartment muscles, respectively. However, in reviewing individual functional (S1 Table) and glycosylation (S1 Fig) data, we saw no such association.

We only assessed one dog (Nani) at the 6X10^14^ dose, so it is difficult to draw conclusions regarding a potential dose effect. However, if anything, levels of functional gain were less pronounced in this dog (Table 1).

Heart function was also assessed at 3, 4½, and 6 months using echocardiography (S2 Table). Comparing data from the 2x10^14^vg/kg *GALGT2* treatment group to the same four untreated GRMD control dogs, no significant differences were found between the two groups (p > 0.05 for all).

On assessment of myocardial T1 mapping in CMR imaging, we compared the extracellular volume (ECV) at 6 months of age. Notably, the ECV of low-dose *GALGT2*-treated GRMD dogs did not differ from untreated GRMD control dogs (25.9±3.4% vs. 22.9±1.9%, n = 4/group). No difference of ECV was seen between untreated control GRMD dogs and wild type GR dogs from another unpublished natural history study in our laboratory (22.9±1.9% vs. 24.2±2.1%, n = 4 and 8 per group, respectively). Thus, ECV was assessed primarily to distinguish versus a treatment effect. No difference of ECV was seen between low-dose *GALGT2*-treated GRMD dogs and wild type GR dogs (25.9±3.4% vs. 24.2±2.1%, n = 4 and 8 per group, respectively). The only high dose treated dog (Nani) had an ECV of 31.5%.

### Assessment of skeletal and cardiac muscle pathology

**Fig 4:**
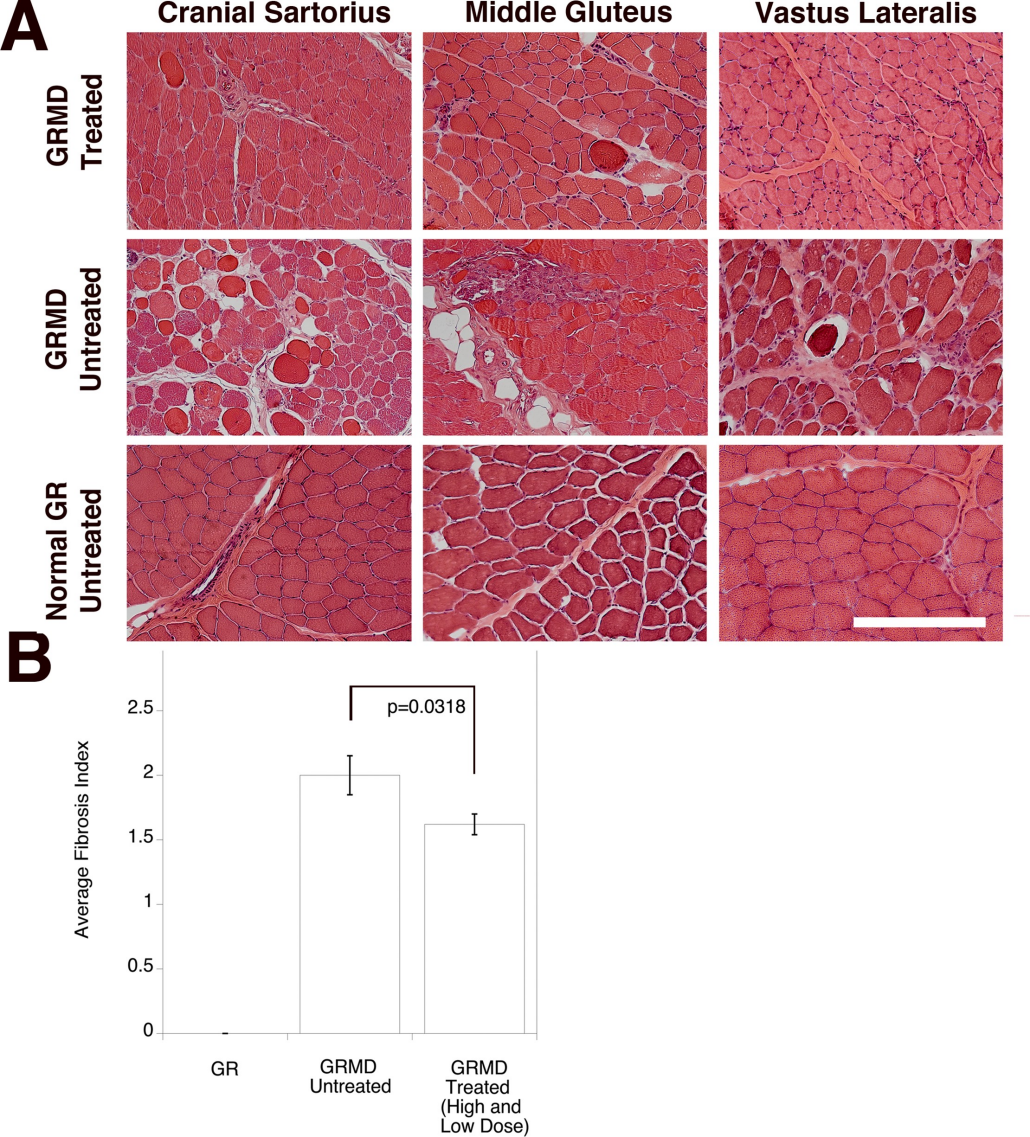
Skeletal muscle pathology in rAAVrh74.MHCK7.*GALGT2*-treated GRMD dogs. (A) Cranial sartorius, middle gluteus, and vastus lateralis muscles were cut in cross-section from 6-month-old untreated normal GR, untreated GRMD, and treated GRMD (treated with low dose, 2x10^14^vg/kg rAAVrh74.MHCK7.*GALGT2* at 3 months) dogs. Sections were stained with H&E. Bar is 200μm. (B) Measures of fibrotic index for skeletal muscles from wild type golden retriever (GR) and golden retriever muscular dystrophy (GRMD) dogs, either untreated or treated (pooled low dose [2x10^14^vg/kg] plus high dose [6x10^14^vg/kg]) with rAAVrh74.MHCK7.*GALGT2*. Fibrotic index (percentage non-muscle area in muscle) measures were based on the following values: 0, no evident fibrosis, 1, 0<x≤5% fibrosis, 2, 5%<x≤15% fibrosis, 3, 15%<x≤25% fibrosis, 4, 25%<x≤50% fibrosis, and 5, x>50% fibrosis. Errors are SEM for n = 9 (GR), 26 (GRMD Untreated), 54 (GRMD Treated Low and High Dose) different muscle biopsies.

We next assessed muscle histopathology by analyzing tissue sections stained with H&E (Fig 4A) and objectively determining the cumulative fibrosis index from biopsied skeletal muscles (Fig 4B). Subjectively, skeletal muscles of treated GRMD dogs generally had less pronounced dystrophic changes than seen in seven combined untreated GRMD dogs (1 from this study and 6 others from previous studies). This was supported by an objective reduction in the relative amount of fibrosis (or non-muscle area in muscle). Individual muscle sections were given a score between 0 and 5, with 0 being no evident fibrosis, 1 for 0<x≤5% fibrosis, 2 for 5%<x≤15%, 3 for 15%<x≤25%, 4 for 25%<x≤50%, and 5 for x>50%. To calculate the overall fibrosis index, we averaged scores in all dogs within each cohort (Fig 4B). Normal, untreated GRs had no evidence of fibrosis, while the combined seven untreated GRMD dogs had an average score of 2.0±0.15 (or between 5 and 15% non-muscle area within muscle). Pooling the four low-dose and one high-dose treated dog samples, we found a mild but significant (p = 0.032) reduction in fibrosis index with treatment, to 1.62±0.08. Thus, fibrosis index was lowered by 19% in treated GRMD muscles relative to untreated GRMD muscles.

Consistent with a lack of functional changes, we saw no significant regions of fibrosis in any untreated or treated (low and high doses) GRMD hearts, with all heart measures showing fibrosis in less than 5% of the total heart area stained (not shown).

### Assessment of immune responses in rAAVrh74.MHCK7.GALGT2-treated GRMD dogs

**Fig 5:**
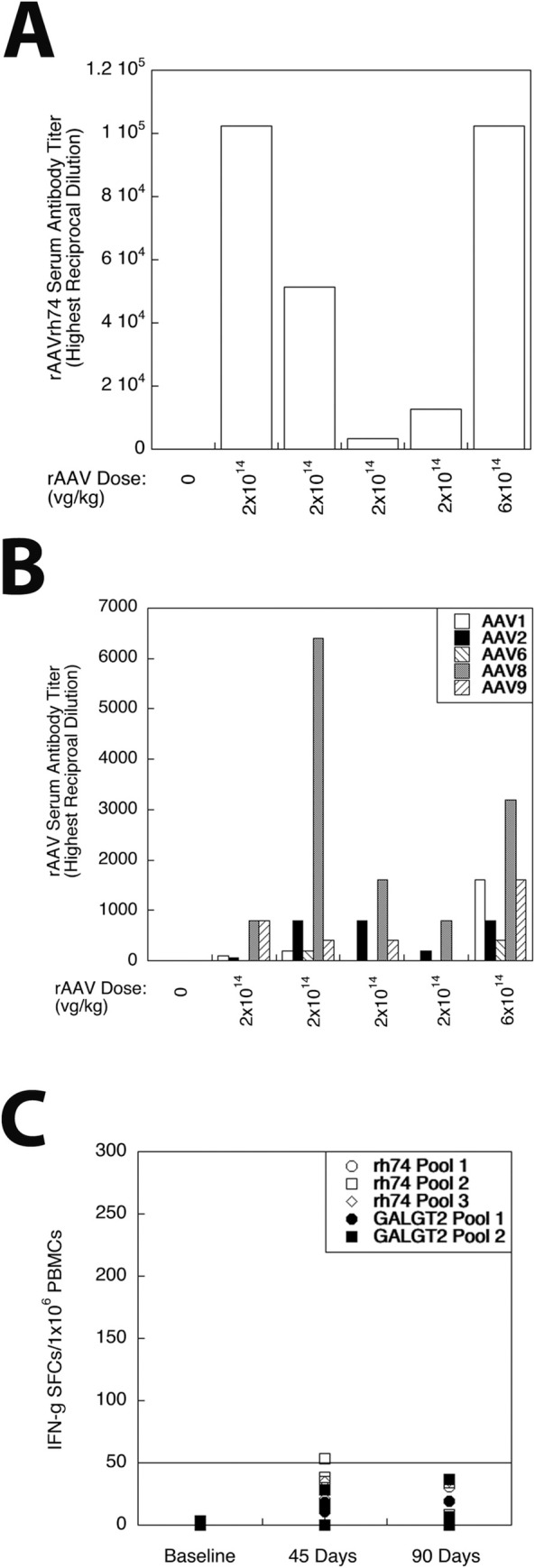
Immune responses to rAAVrh74.MHCK7.*GALGT2* treatment of GRMD dogs. (A) Highest reciprocal dilution at which serum antibodies to rAAVrh74 capsid protein could be detected in one untreated GRMD dog (0 dose), four different GRMD dogs treated with low dose of rAAVrh74.MHCK7.*GALGT2* (2x10^14^vg/kg), and in one dog treated with high dose of rAAVrh74.MHCK7.*GALGT2* (6x10^14^vg/kg). (B) Highest reciprocal dilution at which serum antibodies could be identified to rAAV1, rAAV2, rAAV6, rAAV8, or rAAV9 capsid protein after treatment with rAAVrh74.MHCK7.*GALGT2*. (C) Interferon gamma (IFN-g) expressing cells were quantified in ELISpot assays of peripheral blood mononuclear cells (PBMCs) taken from GRMD dogs at 3 months of age (baseline, pretreatment) and at 45 or 90 days after treatment with rAAVrh74.MHCK7.*GALGT2*. Positive ELISpots to identify activated T cells were measured in response to 1 of 3 overlapping peptide pools made against the rAAVrh74 capsid protein and to 1 of 2 overlapping peptide pools made against the GALGT2 protein. ELISpot scores of 50 or more (line) are considered positive.

We measured serum antibody responses by ELISA to the AAV capsid proteins at 6 months of age, after 3 months of treatment with rAAVrh74.MHCK7.*GALGT2* (Fig 5A and 5B). While no GRMD dogs, treated or untreated, had serum antibodies to rAAVrh74, AAV1, rAAV2, rAAV6, rAAV8 or rAAV9 prior to treatment, all GRMD dogs treated with rAAVrh74.MHCK7.*GALGT2* showed significant elevations in anti-rAAVrh74 antibodies by 3 months post-treatment. Positive assay responses were evident at a dilution ranging from 1:3,200 to 1:102,000 in treated GRMD dogs. As previously observed with intra-arterial treatment of rhesus macaques [62], exposure to the rAAVrh74 capsid also led to production of serum antibodies that could cross-react with other rAAV serotypes. At 3 months post-treatment, 5 of 5 treated GRMD dogs showed positive serum antibody responses to rAAV2 and rAAV8 (with highest positive titers at 1:800 and 1:6,400 dilution, respectively); 4 of 5 dogs had serum antibodies to rAAV9 (with highest positive titer at 1:1,600 dilution); 3 of 5 had antibodies to rAAV1 (with highest positive titer at 1:1,600 dilution); and 2 of 5 had antibodies to rAAV6 (with highest positive titer at 1:400 dilution). There were no serum antibodies identified at a 1:50 dilution against human GALGT2 protein at any time-point in any treated GRMD dog (not shown).

ELISpot assays to peptide libraries against the viral capsid (rAAVrh74) protein and the human GALGT2 protein, which is 82% identical to the canine GALGT2 protein, were measured at baseline, 45 days and 90 days post-treatment (Fig 5C). Interferon-γ-positive spots per million peripheral blood mononuclear cells (PBMCs), a measure indicative of activated T cells, were measured, with 50 or more spots/10^6^ PBMCs considered positive. At baseline, no peptide pool or vehicle alone (DMSO) had more than 5 spots per million PBMCs. At 45 days post-treatment, DMSO alone did slightly exceed 50 spots/10^6^ PBMCs (56 spots) for one treated dog (not shown), and a different treated GRMD dog had a positive signal (of 53 spots) for rAAVrh74 peptide pool 2. No other peptide pool showed positive signals (≥50 spots/10^6^ PBMCs) for any other treated GRMD dog at 45 days, and no peptide pool for any GRMD dog was positive at 90 days. For all assays, Concanavalin A (ConA), a positive control, yielded signals above 50 spots/10^6^ PBMCs (average 285±140 spots at baseline, 194±74 spots at 45 days, and 192±48 spots at 90 days).

### Assessment of serum chemistry, hematology, and non-muscle organ pathology

Serum chemistries and hematology testing was done at baseline and at 45- and 90-days post-treatment (Table 2 and S2 Table, respectively). Some measures were also assessed at 24 and 72 hours. Creatine kinase (CK), aspartate aminotransferase (AST), and alanine aminotransferase (ALT) values were elevated from normal readings at baseline in keeping with GRMD natural history studies [71, 72]. In the four low-dose GRMD dogs, serum CK levels (mean ±SD) were reduced from 38,182 ±17,096 U/L at baseline to 28,231±9,564 U/L at 90 days. However, notably, natural history GRMD CK values showed a comparable decrease [44]. Serum calcium levels increased from 10.9±0.3 mg/dL at baseline to 12.1±1.9 mg/dL at 90 days with the lower 2x10^14^vg./kg dose, but this elevation was not significant (p = 0.18). For the higher dose 6x10^14^vg/kg treated GRMD dog, serum calcium increased further to 14.9 mg/dL. Other serum chemistry and hematologic values in the treated GRMD dogs did not differ from baseline to post-treatment.

**Table 2:** Serum chemistry tests.

Te Test	Normal Range	Baseline	24 Hrs LD	72 Hrs LD	45 Days LD	45 Days HD	90 Days LD	90 Days HD
**TPRO** g/dL	5.6–7.9	5.3±0.4	6.0±2.4	6.1±0.3	5.3±0.2	5.3	5.8±0.1	5.6
**ALB** g/dL	2.8–4.3	2.9±0.2	3.3±1.6	3.3±0.2	2.9±0.2	3	3.1±0.1	3.1
**Ca** mg/dL	7.2–12.8	10.9±0.3	11.2±5.5	11.0±0.4	12.3±2.2	11.3	12.1±1.9	14.9
**PHOS** mg/dL	2.3–6.5	9.5±0.6	9.4±4.6	8.4±0.8	8.7±1.6	8.1	8.7±0.8	7.4
**GLU** mg/dL	60–120	121±9	122±67	123±13	110±14	114	111±9	130
**BUN** mg/dL	8–30	18.7±3.2	16.8±9.0	15.8±3.9	14.8±10.4	7	18.8±1.9	20
**CREA** mg/dL	0.5–1.4	0.6±0.2	0.5±1.5	0.5±0.1	0.4±0.2	0.4	0.5±0.1	0.4
**TBIL** mg/dL	0.1–0.4	0.2±0.0	0.2±1.7	0.2±0.1	0.2±0.1	0.2	0.2±0.1	0.1
**ALP** U/L	12–122	78±12	90±41	84±11	56±14	79	36±5	45
**CK** U/L	40–211	38182±17096	30271±19124	23578±6895	21633±8480	ND	28231±9564	36417
**AST** U/L	13–52	433±176	468±217	343±97	304±101	511	433±132	524
**ALT** U/L	13–79	278±52	349±147	313±78	277±81	382	332±79	469
**GLOB** g/dL	1.8–4.2	2.4±0.3	2.8±1.4	2.8±0.2	2.5±0.1	2.3	2.7±0.1	2.5
**ALB/GLOB** ratio		1.2±0.1	1.2±1.5	1.2±0.1	1.2±0.1	1.3	1.2±0.1	1.2
**GGT** U/L	0–10	<3	<3	<3	<3	<3	<3	<3
GLDH* U/L	0–14	15	15±10	12±13	13±3	10	10±1	10
**Amylase** U/L	200–953	450±25	435±252	436±100	529±49	512	629±65	579
**Cholesterol** mg/dL	124–335	287±34	341±156	321±47	330±28	220	298±31	244
**Na** mEq/L	141–156	143±2	144±81	145±1	146±1	143	146±1	146
**K** mEq/L	3.8–5.5	5.8±0.2	5.8±2.8	5.5±0.3	4.9±0.8	5.7	5.3±0.2	4.5
**Na/K** ratio		24.9±0.4	25.4±13.4	26.6±1.2	30.6±5.4	25.1	27.9±1.4	32.4
**Cl** mEq/L	109–124	107±2	105±60	106±2	106±3	104	107±3	104

Serum chemistry tests from GRMD dogs at baseline (3 months of age) and at 24 hours, 72 hours (Hrs), 45 Days, and 90 Days after treatment with low dose (LD, 2x10^14^vg/kg, n = 4) or a high dose (HD, 6x10^14^vg/kg, n = 1) rAAVrh74.MHCK7.*GALGT2*. Errors are SD for n = 3 (24Hrs, LD) or 4 (all other LDs, save *).

*n = 1-2/grp, ND–not determined.

TPRO-Total (Serum) Protein

ALB-Albumin

Ca-Calcium

PHOS-Phosphorus

GLU-Glucose

BUN-Blood Urea Nitrogen

CREA-Creatinine

TBIL-Total Bilirubin

ALP-Alkaline phosphatase

CK-Creatine Kinase

AST-Aspartate Aminotransferase

ALT-Alanine Aminotransferase

GLOB-Globulin

A/G ratio-Albumin to Globulin

GGT-Gamma Glutamyl Transferase

GLDH-Glutamate Dehydrogenase

Na-Sodium

K-Potassium

N/K ratio-Sodium to Potassium

Cl-Chloride

Non-muscle organs, including liver, spleen, lung, pancreas, kidney, adrenal gland, thyroid, brain (frontal cortex and cerebellum), spinal cord (thoracic and lumbar) and gonads (ovary or testicle) were fixed in formalin, embedded, cut and stained with H&E. For non-muscle organs, we did not see histopathologic lesions in treated GRMD dogs, with the exception of one low dose treated GRMD dog that had a large mononuclear cellular infiltrate in the thyroid (not shown).

### Expression of glycosylated α dystroglycan and upregulation of surrogate genes and proteins

**Fig 6:**
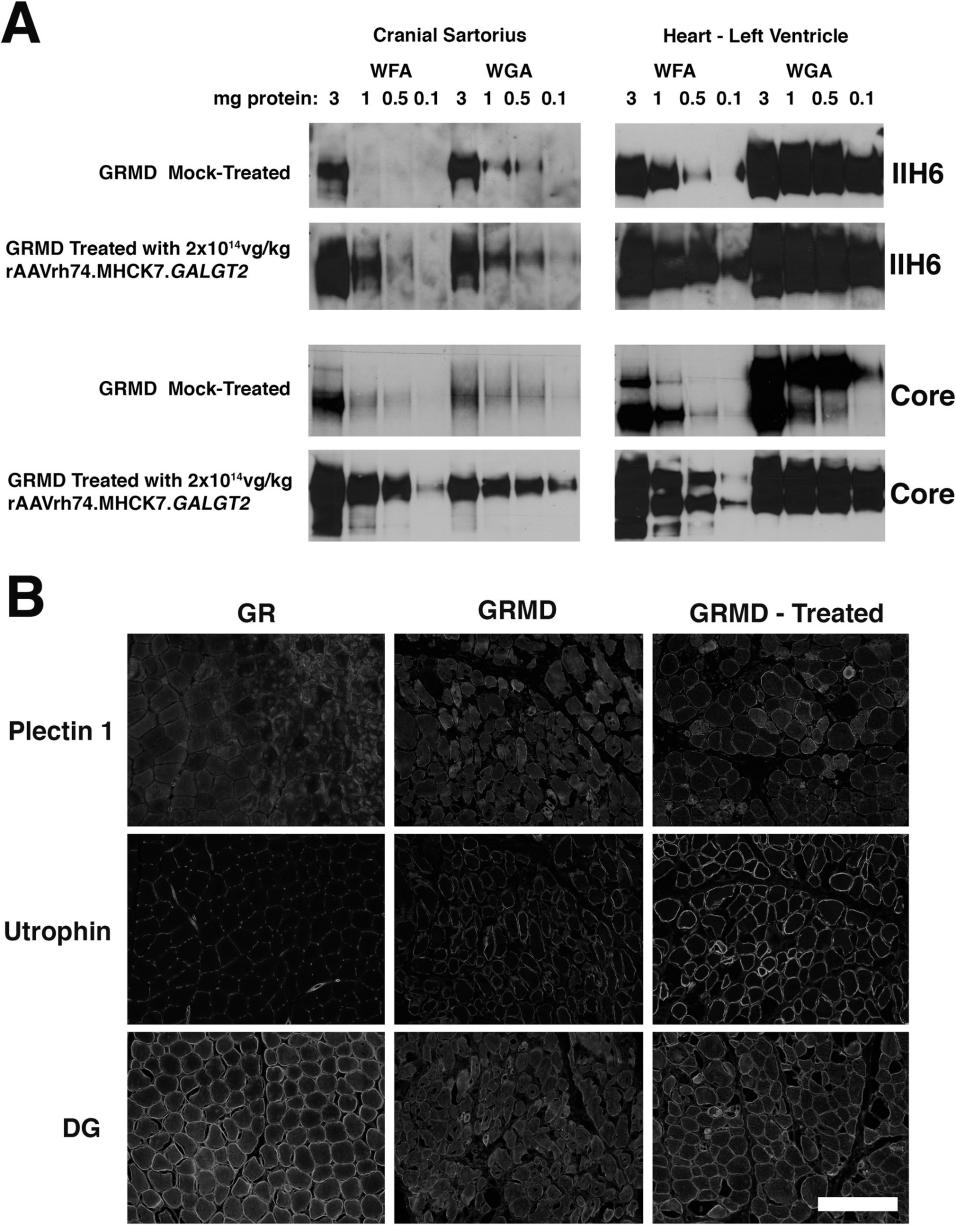
Expression of glycosylated α-dystroglycan and utrophin in response to rAAVrh74.MHCK7.*GALGT2* treatment of GRMD dogs. (A) Lectin precipitations of non-ionic detergent whole protein lysates were completed using different amounts of cellular protein ranging from 0.1- to 3.0mg. Precipitates were solubilized in SDS denaturation buffer, separated by SDS-PAGE, and immunoblotted for α dystroglycan using the IIH6 monoclonal antibody, which recognizes functionally glycosylated αDG, or with antiserum made to the CORE DG polypeptide. The 160kDa region of the gene is shown for IIH6 and the 100-160kDa region is shown for CORE. WFA was used to precipitate α-dystroglycan containing βGalNAc, which is made by GALGT2, while WGA was used to precipitate glycosylated α dystroglycan not containing βGalNAc. (B) Muscle sections taken from the cranial sartorius muscle of normal untreated wild type golden retriever (GR), untreated golden retriever muscular dystrophy (GRMD), or GRMD dogs treated with 2x10^14^vg/kg rAAVrh74.MHCK7.*GALGT2*. Time-matched exposures are shown comparing immunostaining for dystrophin surrogates plectin 1 and utrophin as well as dystroglycan (DG). Bar is 200μm.

In mice and in rhesus macaques, *GALGT2* overexpression induced glycosylation of α dystroglycan (αDG) and increased surrogate gene and protein expression that are known to impact dystrophic pathology, including utrophin and plectin 1 [73, 74]. We therefore analyzed *GALGT2*-depedent changes in αDG glycosylation in rAAVrh74.MHCK7.*GALGT2*-treated skeletal muscle (cranial sartorius) and heart (Fig 6A), as previously described [17]. Both WFA, which binds βGalNAc placed on αDG by *GALGT2*, and Wheat Germ Agglutinin (WGA, which binds sialic acids on αDG that are more accessible when GALGT2 glycosylation is not present), precipitated more αDG in heart than in the CS of untreated GRMDs. Muscle biopsy samples from 6-month-old historical GR and GRMD dogs used for analysis of fibrosis (Fig 4) were used here for lectin precipitation (Fig 6A), immunostaining (Fig 6B), western blot (S3 Fig), and qRT-PCR studies (S4 Fig). These samples were compared to muscle biopsies from untreated GRMD and *GALGT2*-treated GRMD dogs used in this study. For *GALGT2*-treated muscles, the amount of αDG precipitated increased with WGA and WFA relative to muscle lysates from untreated GRMDs at all protein concentrations with positive signals, suggesting increased expression of glycosylated αDG protein. WFA precipitated αDG in the CS muscle when a total protein amount as low as 0.5mg was used, as recognized by IIH6, an antibody to functionally glycosylated αDG, and WFA precipitated αDG when 0.1mg was used when blotted with a polyclonal antisera made to the core DG polypeptide. By contrast, αDG precipitation by WFA was only seen when higher amounts of protein were used in untreated muscle. A similar shift was seen when muscle from the left ventricle of treated and untreated GRMD heart muscle was compared. Both of these results suggest increased muscle expression of glycosylated αDG in rAAVrh74.MHCK7.*GALGT2*-treated dogs.

We next compared protein expression for dystrophin surrogates that can bind DG, including utrophin and plectin 1, by immunostaining (Fig 6B) and for utrophin by immunoblotting (S3 Fig). Results were highly variable between untreated GRMD dogs. For dystroglycan, utrophin, and plectin 1 immunostaining of the CS muscle, some untreated GRMD dogs had high levels of expression and some had low levels (not shown). By contrast, most treated GRMDs had high levels of expression for all three proteins. This was also true for immunoblots, though levels in some treated GRMD muscles were not elevated and some were dramatically elevated compared to untreated GRMDs. Thus, in general, *GALGT2*-treatment elevated surrogate protein expression relative to untreated GRMDs; however, the variable expression in untreated GRMDs made it impossible to draw firm conclusions.

Last, we determined gene expression changes for therapeutic DMD genes in treated and untreated GRMD muscles (S4 Fig). rAAVrh74.MHCK7.*GALGT2*-treated CS muscle showed a 1.5-fold increase in utrophin (Utrn), dystroglycan (Dag1), and dystrophin (Dmd) mRNA relative to untreated GRMD, while there was a 5-7-fold increase in integrin β1 (Itgb1) and laminin α4 (Lama4). For heart, *GALGT2* treatment led to more uniform elevations, with Utrn being elevated 12-fold, integrin α7 (Itga7), Itgb1, Dmd, and Dag1 about 4-fold, and laminin α2 (Lama2), Lama4, and laminin α5 (Lama5) 3-5-fold. Thus, as in mice [9, 39] and rhesus macaques [64], *GALGT2* overexpression induced transcription of therapeutic DMD genes in GRMD dogs.

## Discussion

Our clinical plan for *GALGT2* gene therapy has been to move from intramuscular (IM) injection to isolated limb infusion (ILI) studies, and finally to systemic intravenous (IV) delivery in DMD patients. Pre-clinical IM and ILI studies completed thus far in *mdx* mice and normal macaques [17, 64, 69, 75–77] have allowed us to move to an IND (16175) for a phase 1/2a ILI clinical trial for DMD patients (NCT03333590). We believed, however, that additional IV studies in GRMD dogs were warranted to complement our systemic delivery studies in mice [9, 68, 76] and macaques [62–64, 69, 77] prior to embarking on an IV DMD clinical trial. Here, we tested single dose, systemic delivery of rAAVrh74.MHCK7.*GALGT2* in five GRMD dogs (four dosed at 2x10^14^vg/kg and one dosed at 6x10^14^vg/kg), comparing a range of outcome parameters between baseline at 3 months of age and 3 months after treatment at 6 months. This 3-6-month age range is a period of rapid disease progression, thus offering a relatively short window to establish treatment efficacy [19, 21]. Conclusions were limited by the small number of dogs in this study.

On pathologic assessment, we saw an encouraging, though modest, reduction in the level of fibrosis in skeletal muscles of treated GRMD dogs. GRMD dogs also showed a trend toward improved TTJ extension torque, which has been our primary outcome parameter in other preclinical GRMD studies [19, 21], and less pronounced ECD 3 months after treatment. Both measures, however, failed to reach significance. That we observed no significant improvement in muscle strength in 3-month-old dogs, who already have evidence of disease, suggests treatment might be more effective if administered earlier in the disease course. For instance, multiple intramuscular injections of recombinant laminin-111 protein in GRMD dogs beginning at 2 weeks of age led to both histopathologic and functional improvement at 3 months when comparted to the opposite untreated limb [78]. We were, however, able to demonstrate that *GALGT2* induces muscle glycosylation and utrophin expression in GRMD skeletal muscle and heart.

Considering that GRMD dogs have minimal even subclinical cardiac disease at 6 months of age, we did not expect to see improved cardiac function. With this said, cardiac echocardiographic values between treated and untreated GRMD dogs did not differ, indicating that the rAAVrh74.MHCK7.*GALGT2* treatment had no apparent deleterious effects even when *GALGT2* expression achieved near-saturating levels of glycosylation, as might be expected with an immune response. Speaking further to the heart, there were no differences in ECV on CMR, in the low dose versus control dogs. The only high dose treated dog (Nani) showed a relatively higher ECV value, but no increase of myocardial fibrosis was found on histopathology. Notably, a calculated value from contrast-related T1 mapping on CMR provides a broader measure of cardiac fibrosis than sampling at necropsy [60, 79].

Further, and consistent with previous systemic high dose studies in mice [9, 68] and rhesus macaques [69], we saw minimal changes in serum chemistry and hematology measures, even using IV doses as high as 6x10^14^vg/kg. With that said, serum calcium levels did increase over the course of the study, from 10.9±0.3 mg/dL at baseline to 12.1±1.9 mg/dL at 90 days with the lower 2x10^14^vg./kg dose. For the higher dose 6x10^14^vg/kg treated GRMD dog, serum calcium increased further to 14.9 mg/dL. Although we found no explanation for the hypercalcemia, for example renal disease of hyperparathyroidism [80], this could warrant further investigation.

While the IV doses we used were high and sufficient to induce saturating levels of *GALGT2*-induced glycosylation in the heart, these doses did not induce saturation of glycosylation in skeletal muscles. At the 2x10^14^vg/kg dose, all regions of the heart were transduced with between 1 and 3 vector genomes (vg) per nucleus. Most skeletal muscles, by contrast, were transduced with between 0.25 and 0.5vg/nucleus. *GALGT2*-induced glycosylation in the heart and skeletal muscles paralleled the levels of AAV transduction, with roughly 90% of cardiomyocytes having increased WFA staining versus a more modest range of 20–35% of all myofibers across most skeletal muscles tested. The kinetic lag between the induction of *GALGT2* gene expression in skeletal muscle and optimal *GALGT2*-induced glycosylation, which can be on the order of months [68], may help to account for these differences. The reduced level of dystroglycan expression, the predominant muscle glycoprotein substrate for GALGT2 [17, 35, 81], in GRMD skeletal muscle relative to GR [82] (and relative to GRMD heart) may have also contributed. The use of the MHCK7 promoter, which drives increased cardiac gene expression relative to skeletal muscle when compared to MCK (CK7) [67], likely was another factor.

As in our previous studies in rhesus macaques [69], we found striking muscle to muscle variability in vg transduction with IV delivery of rAAVrh74. AAV transduction was particularly low or variable in the middle gluteal, rectus femoris, biceps femoris, semimembranosus, and medial head of the gastrocnemius muscles. For other muscles, while at least one of the four low dose dogs showed a biodistribution measure of 1 vg/nucleus, the average for the four dogs taken together was significantly lower. The two exceptions were the cranial tibialis muscle and the heart, in which all dogs showed high levels of AAV transduction. Variable vg transduction usually correlated with variable *GALGT2*-induced glycosylation, but vg transduction was reduced relative to glycosylation in some instances. For example, the one GRMD dog dosed with 6x10^14^vg/kg showed >1vg/nucleus in almost all skeletal muscles (except the middle gluteal, rectus femoris, and semimembranosus), yet the percentage of myofibers glycosylated often did not exceed 50%. We did observe elevated immunostaining for dystroglycan, utrophin, and plectin 1 protein in most *GALGT2*-treated GRMD muscles, but this induction was variable from dog to dog and muscle to muscle, as was the expression of these proteins in untreated GRMD dogs. Such variability in treatment-related protein induction likely resulted from varied vascular access of muscle regions during the IV dosing. Variability in staining may also have arisen from damage and loss of GRMD myofibers over the three-month treatment period.

## Conclusions

In summary, these studies of *GALGT2*-treated GRMD dogs build upon previous systemic delivery experiments in mice [9, 35, 68, 75, 76] and rhesus macaques [64, 69, 77]. Our findings support the notion that short-term (3-month) expression of rAAVrh74.MHCK7.*GALGT2* in GRMD dogs may be safe and can induce muscle glycosylation and increased expression of utrophin and dystroglycan, but that such treatments have only a minimal effect on muscle disease pathology and do not significantly change muscle strength. As such, *GALGT2* gene therapy may be better suited to treatments targeting younger individuals prior to severe onset of muscular dystrophy.

## Supporting information

S1 Fig Biodistribution by GRMD dog of AAV vector genomes in muscle and non-muscle organs after rAAVrh74.MHCK7.*GALGT2* treatment. AAV vector genomes were measured in heart and skeletal muscles (A) or in non-muscle organs (B) by qPCR. Asiago, Ricotta, Destiny and Ash were dosed with 2x10^14^vg/kg, while Nani was dosed with 6x10^14^vg/kg. Abbreviations: R (Right), L (Left), IVS (Interventricular Septum), Diaph (Diaphragm), Lat (Lateral) Head Tri (Triceps), Pect (Deep Pectoral), Bic Brach (Biceps Brachii), SDE (Superficial Digital Flexor), Mid Glut (Middle Gluteus), RF (Rectus Femoris), VL (Vastus Lateralis), Cran Sart (Cranial Sartorius), BF (Biceps Femoris), ST (Semitendinosus), SM (Semimembranosus), MG (Medial head, Gastrocnemius), LG (Lateral Head, Gastrocnemius), Cran Tib (Cranial Tibialis), LDE (Long Digital Extensor).
(TIF)

S2 Fig WFA staining of individual GRMD dogs after treatment with rAAVh74.MHCK7.*GALGT2*. Muscle sections from 6-month-old GRMD dogs treated at 3 months of age with 2x10^14^vg/kg (Asiago, Ricotta, Destiny, Ash) or 6x10^14^vg/kg (Nani) of rAAVrh74.MHCK7.*GALGT2* were assayed for *GALGT2*-induced glycosylation. The percentage (%) of myofibers glycosylated by *GALGT2* overexpression, measured by WFA staining, is shown for each dog.
(TIF)

S3 Fig Western blot analysis of protein expression for utrophin after treatment of GRMD dogs with rAAVrh74.MHCK7.*GALGT2*. Immunoblots were performed on whole muscle protein lysates from wild type GR dogs (Tuco, Sleeping Beauty), untreated GRMD dogs (Hickory, Stitch), GRMD dogs treated with 2x10^14^vg/kg of rAAVrh74.MHCK7.*GALGT2* (Ash, Destiny, Ricotta, Asiago), and a GRMD dog treated with 6x10^14^vg/kg rAAVrh74.MHCK7.GALGT2 (Nani). Proteins were separated by SDS-PAGE and immunoblotted with antibodies to dystrophin, utrophin or GAPDH (a control for protein loading and transfer).
(TIF)

S4 Fig Elevated expression of therapeutic genes after treatment of GRMD dogs with rAAVrh74.MHCK7.*GALGT2*. Relative gene expression, assayed by qRT-PCR, was compared in 6-month-old untreated GRMD dogs and 6-month-old GRMD dogs treated with 2x10^14^vg/kg rAAVrh74.MHCK7.*GALGT2*. Fold elevation is reported in treated versus untreated GRMD muscles. Expression was compared in the cranial sartorius muscle (white bars) and in the heart (left ventricle, black bars). Errors are SD for n = 8 per condition.
(TIF)

S1 Table Functional outcome parameters by dog. (TIF)

S2 Table Cardiac functional data from GRMD dogs. (TIF)
