# Supplementary figures and images for: Short-term treatment of golden retriever muscular dystrophy (GRMD) dogs with rAAVrh74.MHCK7.GALGT2 induces muscle glycosylation and utrophin expression but has no significant effect on muscle strength
Source: PLoS One. 2021 Mar 26;16(3):e0248721. doi: 10.1371/journal.pone.0248721 (PMC7997012; doi:10.1371/journal.pone.0248721)

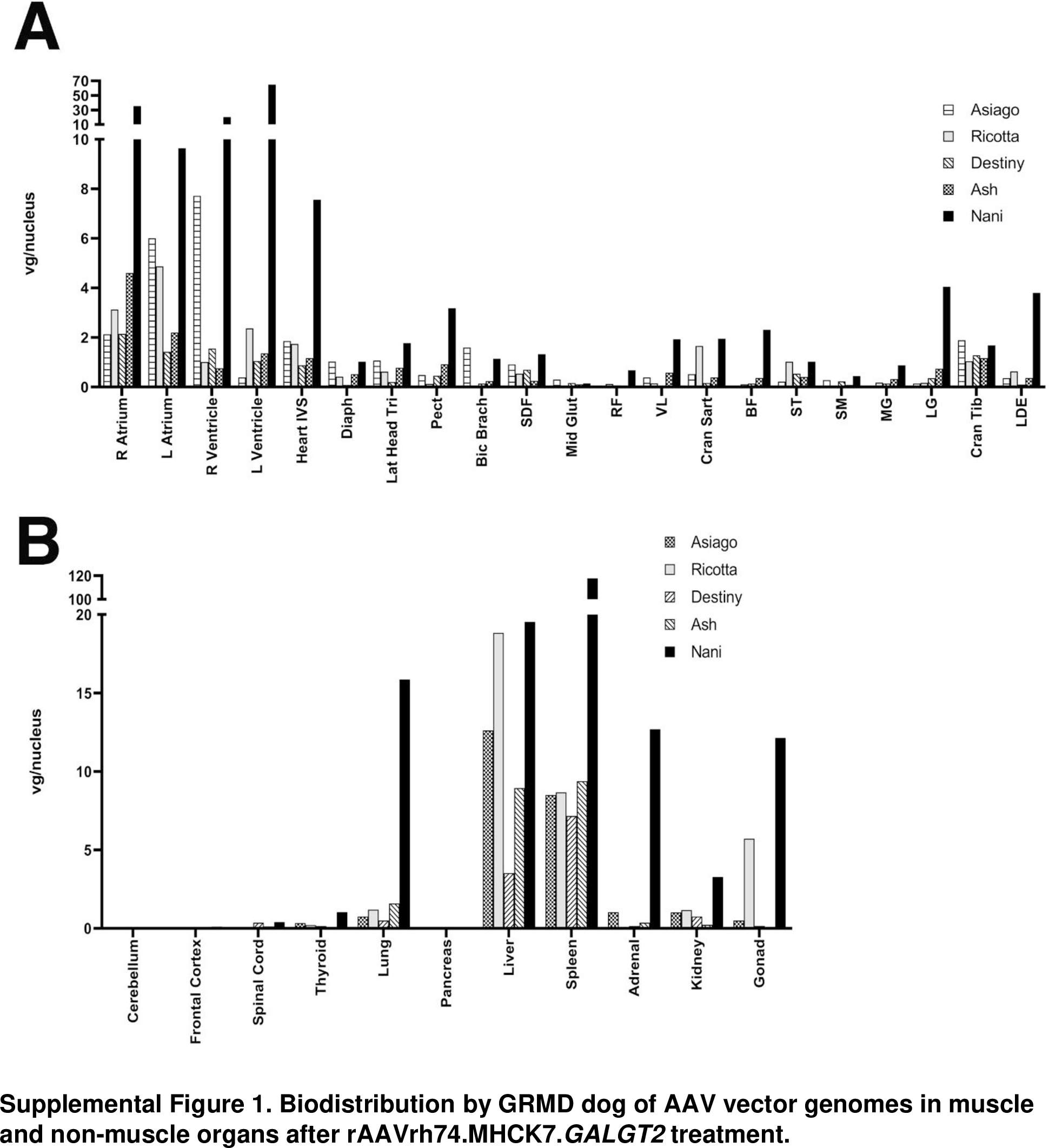

Supplement: S1 Fig — AAV vector genomes were measured in heart and skeletal muscles (A) or in non-muscle organs (B) by qPCR. Asiago, Ricotta, Destiny and Ash were dosed with 2x10^14^vg/kg, while Nani was dosed with 6x10^14^vg/kg. Abbreviations: R (Right), L (Left), IVS (Interventricular Septum), Diaph (Diaphragm), Lat (Lateral) Head Tri (Triceps), Pect (Deep Pectoral), Bic Brach (Biceps Brachii), SDE (Superficial Digital Flexor), Mid Glut (Middle Gluteus), RF (Rectus Femoris), VL (Vastus Lateralis), Cran Sart (Cranial Sartorius), BF (Biceps Femoris), ST (Semitendinosus), SM (Semimembranosus), MG (Medial head, Gastrocnemius), LG (Lateral Head, Gastrocnemius), Cran Tib (Cranial Tibialis), LDE (Long Digital Extensor). (TIF) [file pone.0248721.s001.tif]

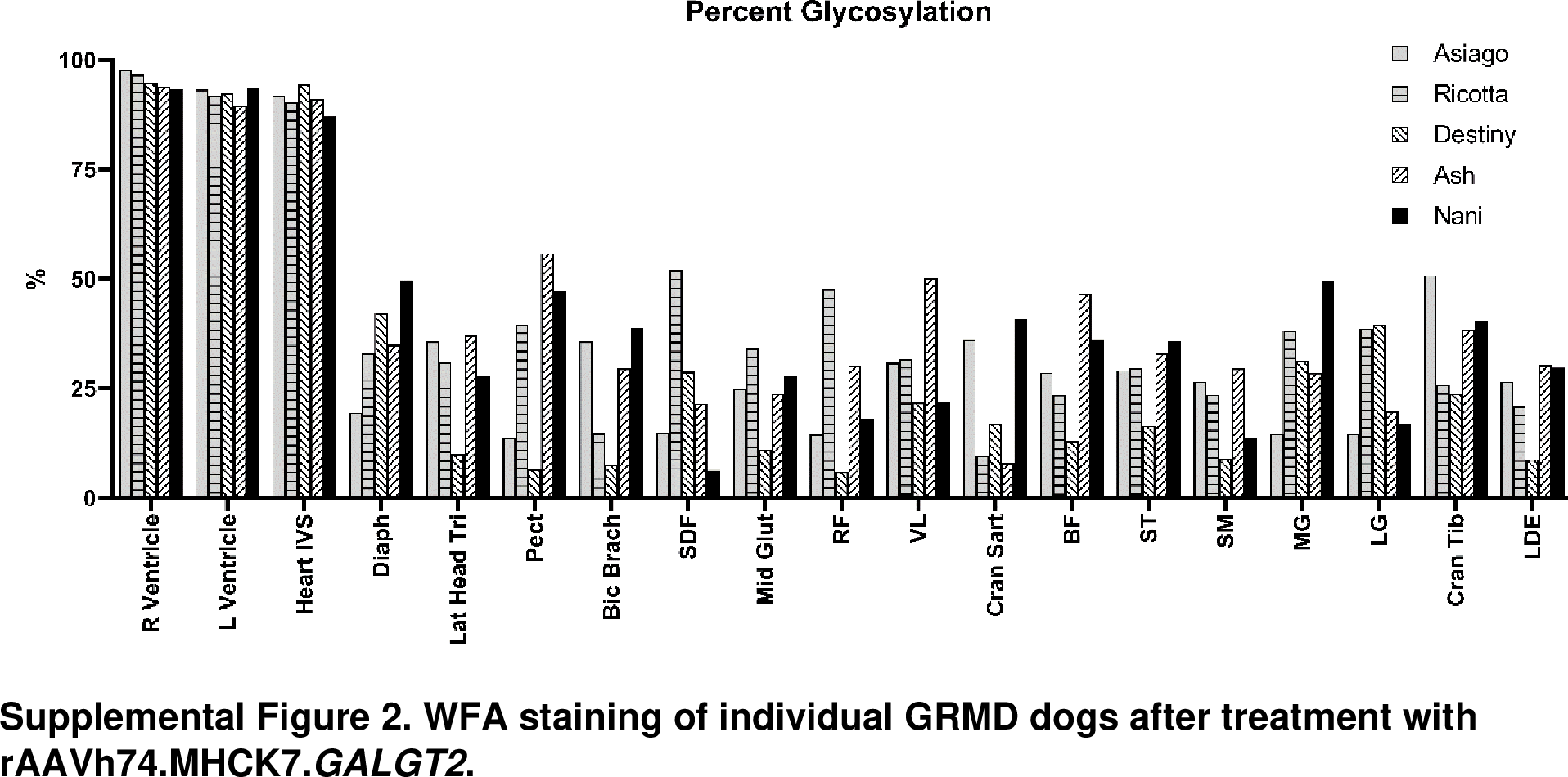

Supplement: S2 Fig — Muscle sections from 6-month-old GRMD dogs treated at 3 months of age with 2x10^14^vg/kg (Asiago, Ricotta, Destiny, Ash) or 6x10^14^vg/kg (Nani) of rAAVrh74.MHCK7.GALGT2 were assayed for GALGT2-induced glycosylation. The percentage (%) of myofibers glycosylated by GALGT2 overexpression, measured by WFA staining, is shown for each dog. (TIF) [file pone.0248721.s002.tif]

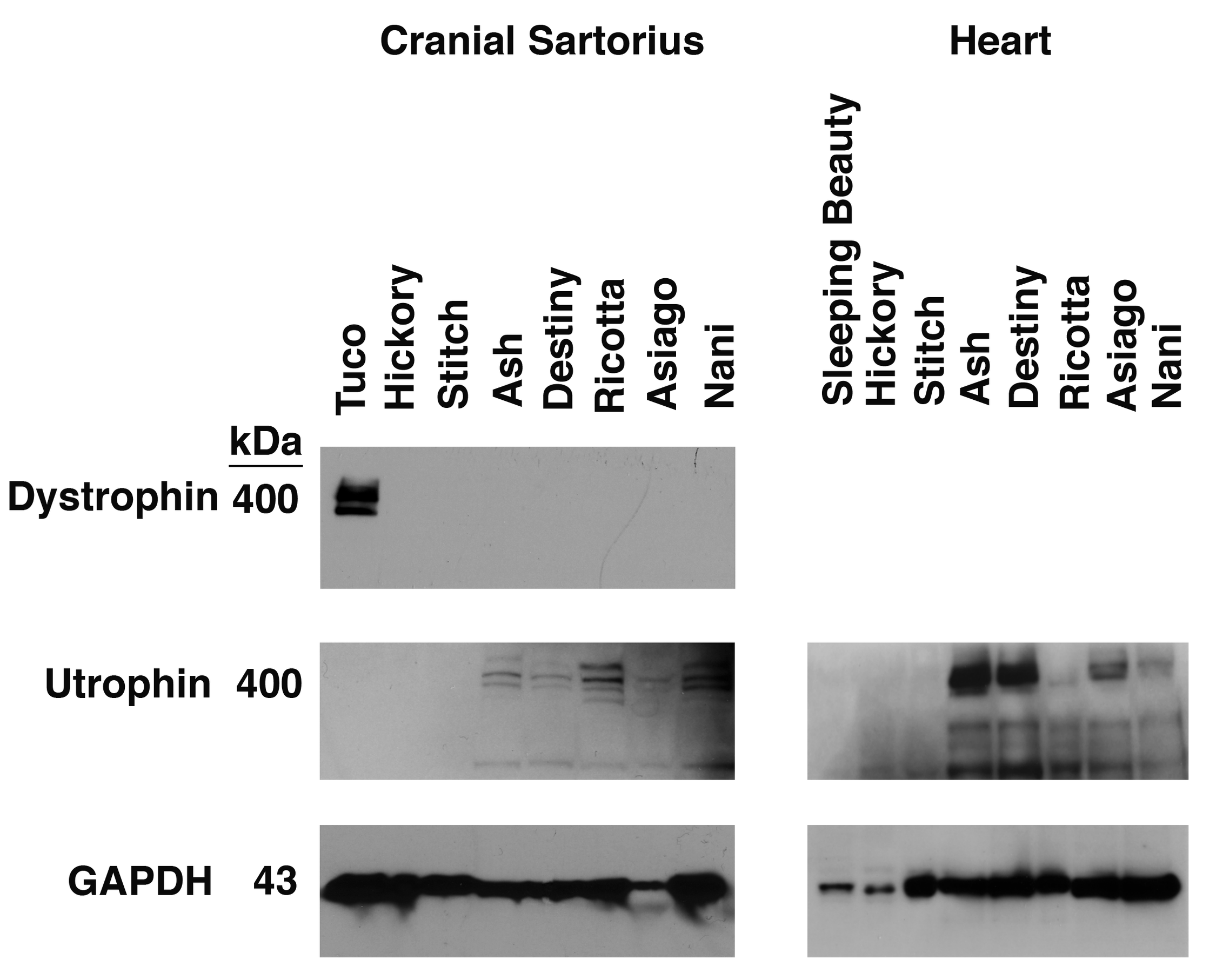

Supplement: S3 Fig — Immunoblots were performed on whole muscle protein lysates from wild type GR dogs (Tuco, Sleeping Beauty), untreated GRMD dogs (Hickory, Stitch), GRMD dogs treated with 2x10^14^vg/kg of rAAVrh74.MHCK7.GALGT2 (Ash, Destiny, Ricotta, Asiago), and a GRMD dog treated with 6x10^14^vg/kg rAAVrh74.MHCK7.GALGT2 (Nani). Proteins were separated by SDS-PAGE and immunoblotted with antibodies to dystrophin, utrophin or GAPDH (a control for protein loading and transfer). (TIF) [file pone.0248721.s003.tif]

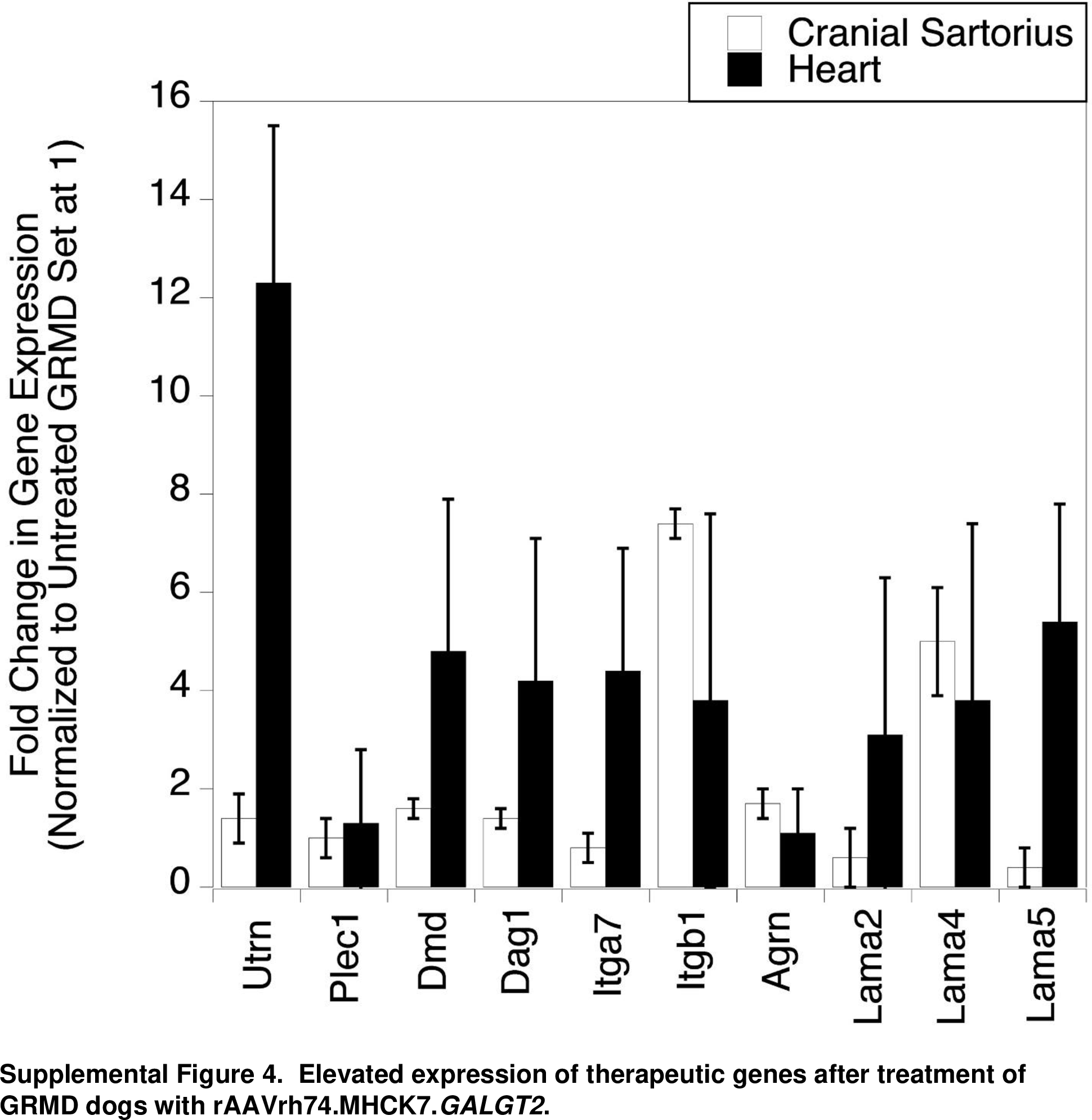

Supplement: S4 Fig — Relative gene expression, assayed by qRT-PCR, was compared in 6-month-old untreated GRMD dogs and 6-month-old GRMD dogs treated with 2x10^14^vg/kg rAAVrh74.MHCK7.GALGT2. Fold elevation is reported in treated versus untreated GRMD muscles. Expression was compared in the cranial sartorius muscle (white bars) and in the heart (left ventricle, black bars). Errors are SD for n = 8 per condition. (TIF) [file pone.0248721.s004.tif]

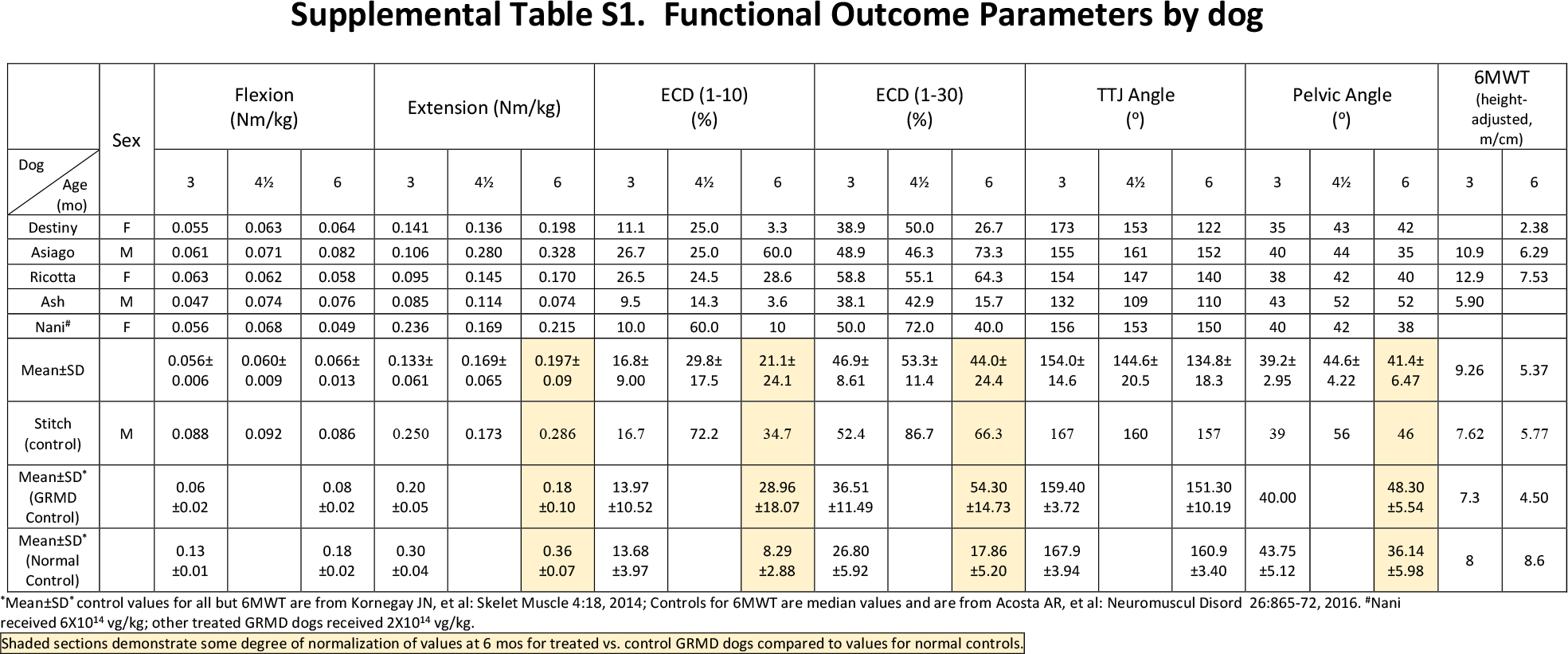

Supplement: S1 Table — (TIF) [file pone.0248721.s005.tif]

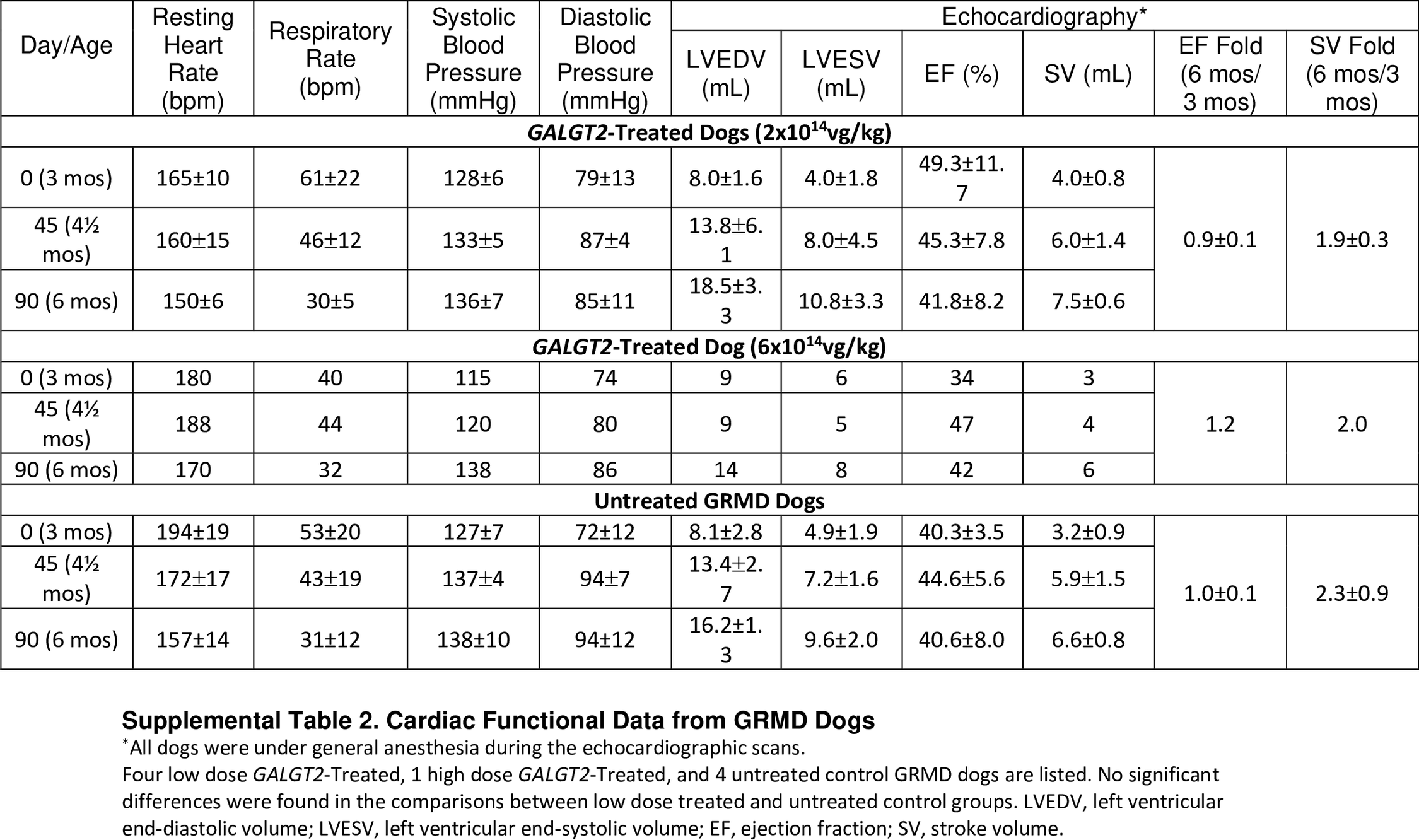

Supplement: S2 Table — (TIF) [file pone.0248721.s006.tif]
